# Antifungal activity against *Fusarium oxysporum* of quinolizidines isolated from three controlled-growth Genisteae plants: structure–activity relationship implications

**DOI:** 10.1007/s13659-023-00373-4

**Published:** 2023-03-20

**Authors:** Willy Cely-Veloza, Lydia Yamaguchi, Diego Quiroga, Massuo J. Kato, Ericsson Coy-Barrera

**Affiliations:** 1 Bioorganic Chemistry Laboratory, Facultad de Ciencias Básicas y Aplicadas, Universidad Militar Nueva Granada, 250247 Cajicá, Colombia; 2 Institute of Chemistry, University of São Paulo, São Paulo, 05508-000 SP Brazil

**Keywords:** Fabaceae, Genista, Lupinus, *Fusarium oxysporum*, Quinolizidines, Antifungals

## Abstract

The Genisteae tribe belongs to the Fabaceae family. The wide occurrence of secondary metabolites, explicitly highlighting the quinolizidine alkaloids (QAs), characterizes this tribe. In the present study, twenty QAs (**1**–**20**), including lupanine (**1–7**), sparteine (**8–10**), lupanine (**11**), cytisine and tetrahydrocytisine (**12–17**), and matrine (**18–20**)-type QAs were extracted and isolated from leaves of three species (i.e., *Lupinus polyphyllus* ('*rusell*' hybrid), *Lupinus mutabilis*, and *Genista monspessulana*) belonging to the Genisteae tribe. These plant sources were propagated under greenhouse conditions. The isolated compounds were elucidated by analyzing their spectroscopical data (MS, NMR). The antifungal effect on the mycelial growth of *Fusarium oxysporum* (*Fox*) of each isolated QA was then evaluated through the amended medium assay. The best antifungal activity was found to be for compounds **8** (IC_50_ = 16.5 µM), **9** (IC_50_ = 7.2 µM), **12** (IC_50_ = 11.3 µM), and **18** (IC_50_ = 12.3 µM). The inhibitory data suggest that some QAs could efficiently inhibit *Fox* mycelium growth depending on particular structural requirements deduced from structure–activity relationship scrutinies. The identified quinolizidine-related moieties can be involved in lead structures to develop further antifungal bioactives against *Fox*.

**Supplementary Information:**

The online version contains supplementary material available at 10.1007/s13659-023-00373-4.

## Introduction

Quinolizidine alkaloids (QAs) are specialized (also called secondary) metabolites biosynthesized from the ʟ-lysine amino acid pathway. They are primarily distributed in the Fabaceae family, one of the largest groups of flowering plants (angiosperms) globally [1]. This family has a cosmopolitan distribution and is well represented in the Colombian flora, being the third largest family among the angiosperms, exceeded by the Asteraceae and Orchidaceae families [2]. QAs are mostly found in some tribes of Fabaceae, such as Genisteae, Sophoreae, Dalbergieae, Euchresteae, Thermopsidae, Bossiaeae, Brongniartieae, Podalyrieae, Liparieae, and Crotalarieae [3]. In addition, QAs have also been identified in numerous series of petrosins, xestospongins, and araguspongins from marine sponges belonging to the genera *Petrosia*, *Xestospongia*, and *Oceanapia* [4], as well as in frogskins, specifically in the Dendrobatidae and Mantellidae families, highlighting some species such as *Phyllobates aurotaenia, Melanophryniscus moreirae, Melanophryniscus toads* [5], *Epipedobates tricolor* [6], *Mantella baroni* [7] and *Mantella basileo* [8]. In the case of plant species, the QAs mainly occur in seeds, pods, leaves, flowers, aerial parts, and roots of the *Lupinus* genus. The *Lupinus*-derived QAs can cause toxicity due to lupanine, lupinine, and hydroxylupanine since they have an excitatory effect on the CNS and a depressant effect on the respiratory and vasomotor centers, including acute anticholinergic toxicity. Consequently, the lupanine derivative-rich seeds must be debittered before human and animal consumption for QAs’ removal.

QA-rich extracts have been reported in the literature for their antimicrobial properties against pathogens such as *Fusarium solani* [9], *Alternaria solani* [10]*,* and *Rhizoctonia solani* [11]. In fact, QAs are considered a plant chemical defense against biotic pressures [12–14]. However, despite the promising antifungal effects of QA-containing extracts, the number of records on the antifungal activity of isolated QAs against phytopathogens is limited, especially on *Fusarium oxysporum* (*Fox*), a local fungus responsible for significant losses in the agricultural and productive sectors [15]. *Fox* is responsible for losing more than 50% of economically important crops worldwide, such as tomatoes, carnations, cape gooseberries, and bananas [16, 17]. The first-line strategy against *Fox* is chemical control, using commercial fungicides that cause resistance, residuality, and soil alterations [18, 19]. In this context, examining natural sources to find antifungals is highly required to be employed for phytopathogen control involving the lowest adverse effects on the environment. Therefore, based on these facts, the present study aimed to isolate QAs from three plants (i.e., *Lupinus polyphyllus* ('*rusell*' hybrid), *L. mutabilis,* and *Genista monspessulana*) belonging to the Genisteae tribe, and propagated under semi-controlled conditions, and evaluate their in vitro antifungal activity against the phytopathogen *Fox*. Finally, some structure–activity relationship considerations were disclosed and discussed for this mid-size set of isolated QAs.

## Results and discussion

### Isolation and characterization of isolated quinolizidines 1–20

After a phytochemical investigation of the selected plant sources, twenty QAs (**1–20**) were extracted under an acid-based procedure and isolated using sequential separation and purification steps through column chromatography. Compounds **1–20** were isolated from three genistoid species, specifically from two *Lupinus* plants (i.e., *L. polyphyllus* ('*rusell*' hybrid) and *L. mutabilis*) and one *Genista* plant (i.e., *G. monspessulana*), which were propagated under greenhouse conditions (see experimental section). Once the QAs were isolated, their structures were determined by the comprehensive analysis of NMR, GC-EI-MS, and HRESIMS data. Hence, the isolated QAs were found to be known compounds, reported in previous phytochemical studies on Fabaceae species, whose data related to GC-based purity, EIMS, HRESIMS, NMR, and optical rotation of **1–20** are included in the supplementary material (Figs. S2–S21). By comparing the spectroscopical data with those reported in the literature, they were elucidated as (–)-lupanine (**1**) [20], ( +)-11,12-dehydrolupanine (**2**) [21], ( +)-5,6-dehydrolupanine (**3**) [22], (–)-4α-hydroxylupanine (**4**) [23], (–)-13α-hydroxylupanine (**5**) [23], (–)-17-oxolupanine (**6**) [24], (–)-α-isolupanine (**7**) [25, 26], (–)-sparteine (**8**) [20], ( +)-aphylline (**9**) [25, 26], (–)-multiflorine (**10**) [27, 28], (–)-lupinine (**11**) [20, 29, 30], (–)-cytisine (**12**) [31], (–)-*N*-methylcytisine (**13**) [31, 32], (–)-*N*-formylcytisine (**14**) [31, 33], (–)-anagyrine (**15**) [34], (–)-tetrahydrorhombifoline (**16**) [35], (–)-angustifoline (**17**) [35, 36], (–)-matrine (**18**) [37, 38], ( +)-lehmanine (12,13-dehydromatrine) (**19**) [39] and (–)-oxymatrine (**20**) [38]. This study led to obtaining six QA types such as lupanine (**1–7**), sparteine (**8–10**), lupinine (**11**), cytisine (**11–15**), tetrahydrocytisine (**16- 17**), and matrine (**18–20**)-type QAs. The structures of isolated compounds are also presented in the supplementary material (Fig. S1). Despite the isolated compounds being known, the present study reports their isolation from greenhouse-propagated *Lupinus* and *Genista* species for the first time, and most of them were first evaluated against *Fox*.

### Antifungal activity of isolated quinolizidines

**Fig. 1:**
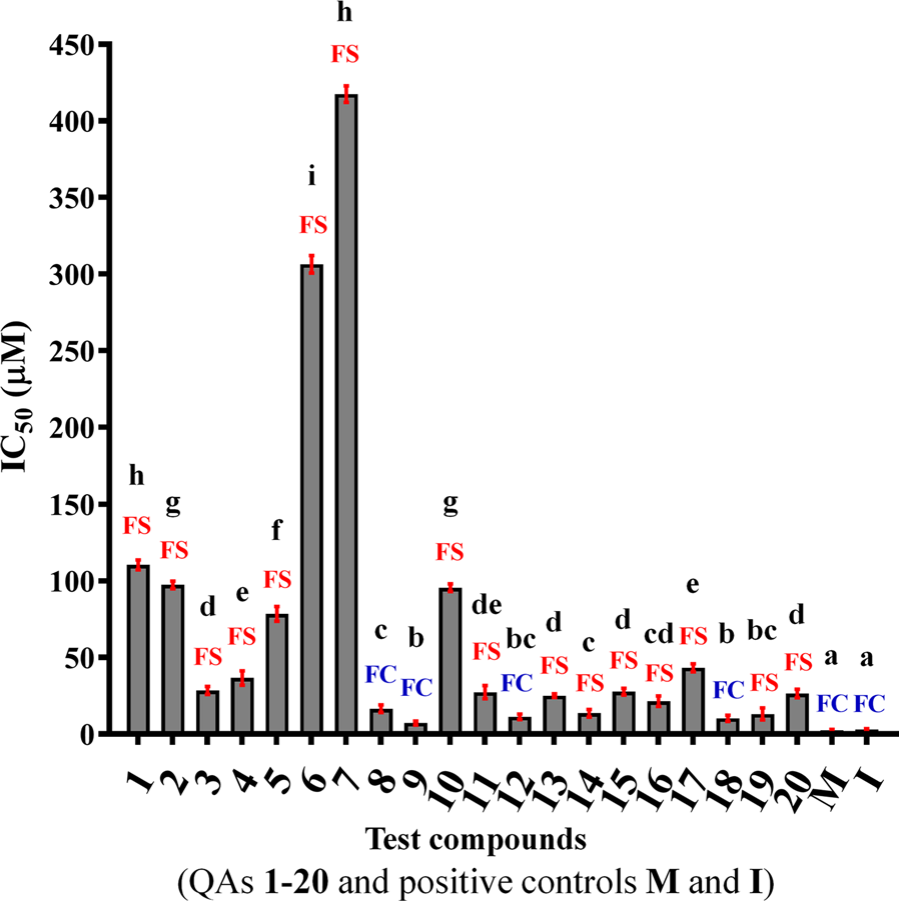
Half-maximal inhibitory concentration (IC_50_ in µM) of test QAs **1**–**20** and positive controls **M** (mancozeb, IC_50_ = 2.45 µM) and **I** (iprodione, IC_50_ = 2.88 µM). Data are expressed as means ± standard deviation (SD) from triplicates. Uppercase colored letters over bars are related to the final effect according to the abbreviations: *FS*  Fungistatic and *FC  *Fungicide. Different lowercase letters over bars represent the statistically significant differences according to the posthoc Tukey test's multiple comparisons (*p* < 0.05)

Compounds **1–20** were evaluated against *Fox* through an amended medium assay to observe their effects on fungal mycelial growth as antifungal action. This effect was assessed using concentrations between 1000 and 0.1 µg/mL. The results were expressed as half-maximal inhibitory concentration (IC_50_ in µM), as shown in Fig. 1.

Antifungal results were classified as most active when IC_50_ was closer to that of the positive controls and least active when the IC_50_ was far from those of the positive controls. In this way, the following classification was then proposed: group **I**: IC_50_ < 15 µM (most active), group **II**: 16 µM < IC_50_ < 50 µM (active), group **III**: 51 µM < IC_50_ < 100 µM (moderately active), and group **IV**: IC_50_ > 100 µM (least active). According to the above results, compounds **8**, **9**, **12,** and **18** comprise group **I** (most active), while compounds structurally related to **1** and **16** were mainly distributed in groups **II-IV** (less active). Furthermore, compounds were also classified as fungistatic (FS) or fungicidal (FC) through an additional experiment after the QA-amended media assay, using the treated mycelium, and transferred to a non-amended fresh medium to observe further growth. FS and FC were defined for every test QA if additional growth or no growth, respectively, was observed. This additional QA classification is advantageous for different purposes since the FC-featured compounds have a role as fungal killers (i.e., fungicides) by stopping the fungal growth completely, even after removing the chemical agent presence, so the regrowth by transferring to non-amended fresh medium is not achievable. In contrast, FS-classified QAs can block the fungal growth during a time-limited exposure to the chemical agent, and consequently, if the chemical pressure is removed (i.e., non-amended fresh medium), the fungus can reactivate its growth [40]. In this regard, the FC effect can be considered more attractive since the fungal presence can be totally suppressed, but the fungicidal/fungistatic effect translation to clinical and field practices can depend on several factors, such as aim/purpose, inoculum, titer, exposure time, administration way, and employed dose [40, 41].

Those alkaloids structurally related to compound **1** (i.e., lupanine-type QAs, compounds **1–7**) exhibited different IC_50_ values within the 28–418 µM range. Compound **3** was the most active lupanine-type QA (IC_50_ = 28.5 µM), while compound **7** was the least active (IC_50_ = 417.5 µM). The base core compound **1** (lupanine) was previously evaluated against *Fox* at 5 µg/µL but did not inhibit the fungal mycelial growth (inhibition = 0%) [42]. This previously-reported outcome coincides with the low activity found in our study, although it did show significant inhibition of mycelial growth on *Sclerotium rolfsii* (89.5%) [42]. The other lupanine-type alkaloids **2–7** have not been evaluated previously against *Fox*. However, some of these QAs, such as **7**, were part of a *Lupinus exaltatus* alkaloidal extract, which was also evaluated at the same concentration of compound **1** (i.e., 5 µg/µL) against *S. rolfsii*, *A. solani*, *R. solani*, and *Fox* [42]. Their inhibition percentages were > 91% for the phytopathogens evaluated, except for *Fox*, which was not inhibited [42]. The antimicrobial properties of *Genista vuralii* extract containing some lupanine-type QAs (e.g., **1**, **3**, and **6**, among other QAs) were reported against *Staphylococcus aureus, Bacillus subtilis, Escherichia coli, Klebsiella pneumoniae, Pseudomonas aeruginosa, Candida albicans,* and *Candida krusei*, with MIC values within 60–500 µg/mL range, and the most susceptible microorganism was *C. krusei* with a MIC = 62.5 µg/mL [43, 44]*.* On the other hand, the *Retama monosperma* alkaloidal extract (containing QAs **2**, **3**, and **7**, among other QAs) had already been reported as not very active in previous antifungal studies against *C. albicans*, *Candida tropicalis,* and *Aspergillus niger* (IC_50_ < 100 µM) [45]. However, a *Lupinus albus* genotype (‘*Multitalia 4*’), containing high contents of **5** (730.9 ± 10.7 mg/kg), exhibited good activity against *K. pneumoniae* (MIC = 16 µg/mL) [14]. Indeed, compound **5** exhibited good activity against *A. niger* by the TLC-bioautography method, inhibiting the fungal growth within four days of the assay [43, 44]*.* A recent study reported that QA **5** was statistically recognized as a relevant phytocomponent contributing to the antifungal action against *Fox* of an optimized alkaloidal extract from *L. mutabilis* leaves [46].

In the case of compounds **8**–**10** (sparteine-type QAs), their IC_50_ values fell into group **I**, especially **8** and **9**, which had IC_50_ < 16 µM, involving a fungicidal effect. Compound **10** had activity corresponding to group **II** and showed a fungistatic effect. Compounds **9** and **10** were herein tested individually against *Fox* for the first time. Only a previous study reported the activity of sparteine (**8**) against a *Fox* strain, but there was no inhibitory activity on fungal growth between 1 and 50 mM [47]. Additionally, **8** and **9** had already shown antimicrobial properties against *S. aureus* ATCC 25,923, *B. subtilis* ATCC 6633, *E. coli* ATCC 25,922, *P. aeruginosa* ATCC 27,853*, C. albicans ATCC 10,231,* and *C. krusei* ATCC 14,243, with a MIC range between 31.25 to 62.5 µg/mL [48]. Compound **11** (IC_50_ = 95.53 µM, fungistatic effect) was the only bicyclic QA obtained in our study. Antifungal activity fell into group **III** and is not comparable with compounds **8** and **9** (< 20 µM). However, compound **11** had antecedents of bactericidal and antifungal capacity when found in high amounts with other QAs such as sparteine, cytisine, ammodendrine, lupanine, and/or feruloylupinine [14, 49].

The highest activity for those QAs structurally related to cytisine was found for **12** (IC_50_ = 11.3 µM, group **I**), involving a fungicidal effect, whereas compounds **13**–**15** showed a fungistatic effect. QA **14** was highlighted because the inhibition values correspond to the group **I** (IC_50_ < 15 µM), while **13** and **15** were classified into group **II**. No antifungal antecedents were found against *Fox* (or other *Fusarium* variants) of compounds **13–15**. However, a crude alkaloidal extract containing compounds **12** and **13,** obtained from *Calia secundiflora* [syn. *Sophora secundiflora*], and the isolated QA cytisine (**12**), showed antimicrobial properties at concentrations between 1 and 6 µg/µL against *A. solani*, *Monilia fruc*ticola, *Fox*, *Xanthomonas campestris*, *Pseudomonas sp.,* and *Erwinia carotovora*, involving significant mycelial growth inhibition diameters (> 5 mm at 6 mg/mL) [49]. Moreover, compounds **12**, **13,** and **15** have also been reported in an alkaloidal extract from *G. vuralii* and evaluated against *E. coli, P. aeruginosa, B. subtilis, S. aureus, C. albicans,* and *C. krusei*, with MIC values within 62.5–500 µg/mL range [43]. Compound **15** has no antifungal records as a pure substance; however, extracts of *Retama raetam* containing compound **9** (43.6%) and **15** (28.5%) mostly showed antimicrobial activity at 75 µg/mL against *S. aureus* (MIC = 125 µg/mL) [50]. Similarly, extracts containing compounds **16** and **17** (tetrahydrocytisine-type QAs) had also shown antimicrobial effects against *E. coli, B. subtilis*, *S. aureus*, *C. albicans*, *C. krusei,* involving a MIC = 128 mg/mL for *P. aeruginosa* and a MIC = 16 µg/mL for *K. pneumoniae* [14, 51]. Additionally, the antibacterial and antifungal activity of the alkaloid extract of *L. angustifolius* containing compounds **16** and **17** were tested against strains of the following bacteria: *E. coli* (MIC = 500 µg/mL), *P. aeruginosa* (MIC = 62.5 µg/mL), *B. subtilis* (MIC = 62.5 µg/mL), and *S. aureus* (MIC = 62.5 µg/mL) as well as the fungi *C. albicans* (MIC = 500 µg/mL) and *C. krusei* (MIC = 500 µg/mL) [51].

Finally, QAs structurally related to **18** (matrine-type) showed a high inhibition against the phytopathogen *Fox*. Compound **18** showed group-**I**–related antifungal activity (IC_50_ < 15 µM), including a fungicidal action. Similarly, compounds **19** and **20** also exhibited inhibition results corresponding to group **I**, but both promoted a fungistatic effect. Matrine-type QAs have been widely referred to in the literature for their antimicrobial properties against phytopathogens. For instance, QAs **18** and **20** showed conidia germination inhibition against *Fox* (EC_50_ = 133 and 26 µg/mL, respectively)*, Cladosporium oxysporum* (EC_50_ = 272 and 573 µg/mL, respectively), and *Marssonina brunnea* (EC_50_ = 123 and 601 µg/mL, respectively) [52], being compound **20** more active than **18**. However, in our case, compound **18** showed the best inhibitory action on *Fox* mycelial growth (IC_50_ = 10.28 µM, FC), and this information agrees with the reported antifungal activity against *C. albicans* [53], *Microsporum lanosum* [54], *Fox*, *Valsa pini*, *Cladosporium oxysporum*, and *Marssonina brunnea* [52]. Compound **20** did not agree with the literature since it has been reported as an antifungal against *Fox*, involving better EC_50_ values, i.e., 26 µg/mL, compared to **18** (123 µg/mL) [52]. However, this apparent inconsistency can be rationalized since the previous study evaluated the antifungal activity through conidia germination inhibition, and our study was oriented to the inhibitory action on mycelial growth as the antifungal effect.

### Structure-activity relationships

**Fig. 2:**
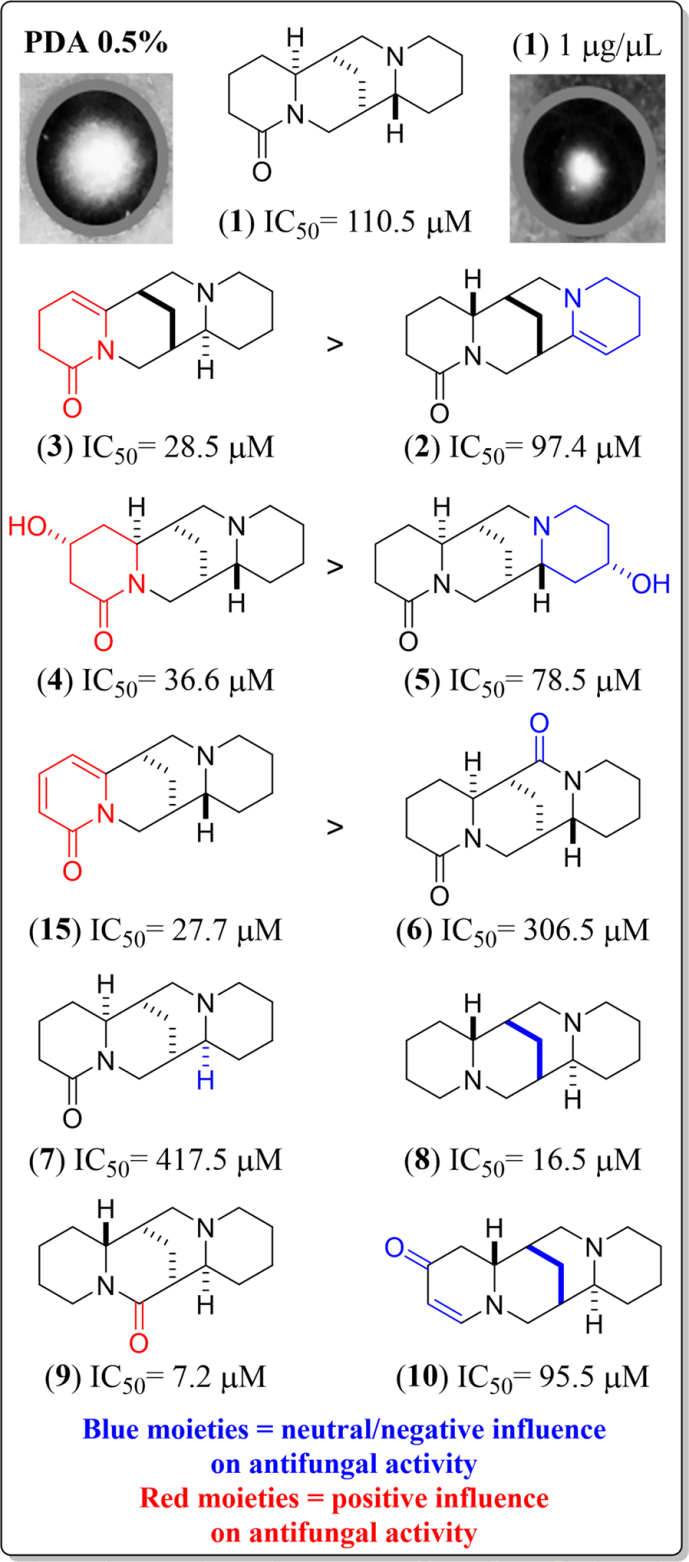
Structure–activity relationships of lupanine- and sparteine-related diazatetracyclic QAs. Blue and red moieties were deduced to have a neutral/negative or positive influence on antifungal activity against *F. oxysporum* compared to QA **1**

The QA’s structural and bioactivity variations of each compound against *Fox* caught our attention. Hence, the relationships between structure and antifungal activity were examined within alkaloid types using the outcome of this mid-size set of isolated QAs. In this regard, for instance, the lupanine-type diazatetracyclic QAs exhibited an antifungal effect at different levels. Such an activity appeared to be influenced by particular structural variations based on substituents and double bonds in different positions, as shown in Fig. 2. The best antifungal activity within the lupanine-type group was found for compound **3** (IC_50_ 28.5 µM, group **II**) and compared to the basic structure related to compound **1** (IC_50_ 110.5 µM, group **IV**). This observation suggests that unsaturations in the A-ring of the QA and the opposite C7-C8-C9 bridge orientation might be critical to obtaining a better antifungal effect against *Fox*. However, a double bond in the D-ring did not show any relevant effect since compound **2**’s IC_50_ (*ca.* 100 µM) was higher than that of **3**. These facts showed the particular influence of the relative bridge orientation and the unsaturation location in the QA structure, i.e., neutral and positive influence if a double bond occurs in the D-ring or the A-ring, respectively. Similarly, compound **15** (an anagyrine-type QA) had a conjugated system in the A-ring (i.e., an α-pyridone moiety) and even exhibited an IC_50_ < 28 µM (group **II**). In addition, compounds **4** and **5** are structurally related to compound **1** but differ by hydroxyl groups at C4 and C13, respectively. In this way, compound **4** had antifungal activity corresponding to group **II**, being more active than compound **5**, which was moderately active (type **III**). Therefore, unsaturations or hydroxyl substituents on the A-ring of lupanine-type QAs were relevant structural factors for better *Fox* growth inhibition. Additionally, a keto group at Cring at C17 (e.g., **6**) and the α-orientation of the H-11 (e.g., **7**) seemed to influence the activity of QAs against *Fox* negatively, being **6** and **7** the least active QAs in this small test compound set (IC_50_ > 300 µM). In contrast, a carbonyl group at C10 (B-ring) instead of C2 (A-ring), and even the opposite orientation for H-6 and H-11 compared to **1**, exhibited a 15-fold improved activity, constituting QA **9** as the most active compound (IC_50_ = 7.2 µM). Finally, the opposite orientation of C7-C8-C9 bridge and the absence of the carbonyl group at C2 for the sparteine-type QAs revealed a *ca.* seven-fold better activity (e.g., **8**, IC_50_ = 16.5 µM). However, a carbonyl group at C4 and a Δ^2(3)^ unsaturation exhibited a neutral influence on the antifungal activity of **10** (IC_50_ = 95.5 µM) compared to **1** but a lesser effect than** 8**.

**Fig. 3:**
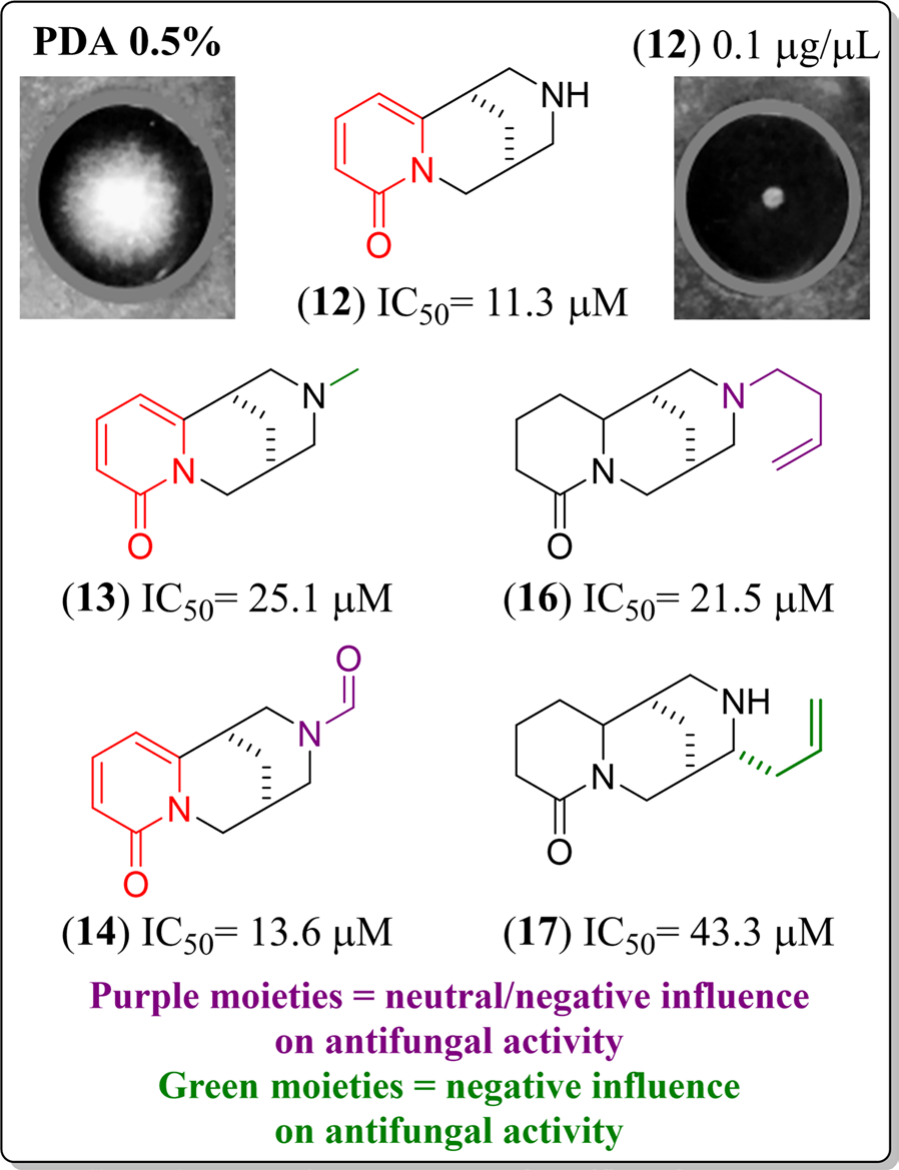
Comparison of *F. oxysporym* mycelial growth inhibition of cytisine-type diazatricyclic QAs. Purple and green moieties were deduced to have a neutral/negative or negative influence on antifungal activity against *F. oxysporum* compared to QA **12**

On the other hand, compounds structurally related to **12** (IC_50_ = 11.3 µM, FC) showed mycelial growth inhibition changes depending on the substitutions at N12 (Fig. 3). For instance, QA **13** had an electron-donating group (EDG, i.e., methyl) at N12, but the activity decreased and was situated into group **II**. In contrast, compound **14** had an electron-withdrawing group (EWG, i.e., formyl) at N12, showing an IC_50_ value corresponding to the group **I**. This fact suggested that the substitution nature on the N12 of cytisine-type QAs is a critical feature for the *Fox* mycelial growth inhibition. Although few molecules related to **12** were isolated in the present study, it was possible to conclude that EWG at N12 can be responsible for group-**I**-related inhibitory effects (most active). In contrast, EDG at N12 can be considered responsible for decreasing the antifungal activity until lower mycelial growth inhibition (**II**, **III**, and **IV** groups).

Furthermore, tetrahydrocytisine-type QAs (i.e., **16** and **17**) also showed an interesting relationship between antifungal activity and the presence of an alkenyl substituent (but-3-enyl or allyl) at N12 or C11, respectively, as shown in Fig. 3. In this regard, compounds **16** and **17** belong to group **II**, but **16** exhibited better activity than **17**, indicating a better influence on the antifungal activity if alkenyl group is positioned at N12 but lower than QA **12**. To expand this observation, a comparison between **13** and **16**, differing by a 2-pyridone moiety and the N12 substituents, can rationalize the structural effect on the antifungal activity of these tricyclic QAs. Compound **13** has a methyl group, while compound **16** has the but-3-enyl moiety. Thus, it was observed that the but-3-enyl substitution increased the inhibition, which suggests that bulkier substitutions at N12 have a positive influence. In contrast, compound **17** contained an allyl substitution at C11, which did not influence the antifungal activity since the IC_50_ was reduced by more than 50% compared to compound **16**. Thus, according to these observations, a short alkenyl chain at C11 does not contribute to the better antifungal activity. Contrarily, a positive impact can be deduced if a but-3-enyl substitution is attached at N12.

**Fig. 4:**
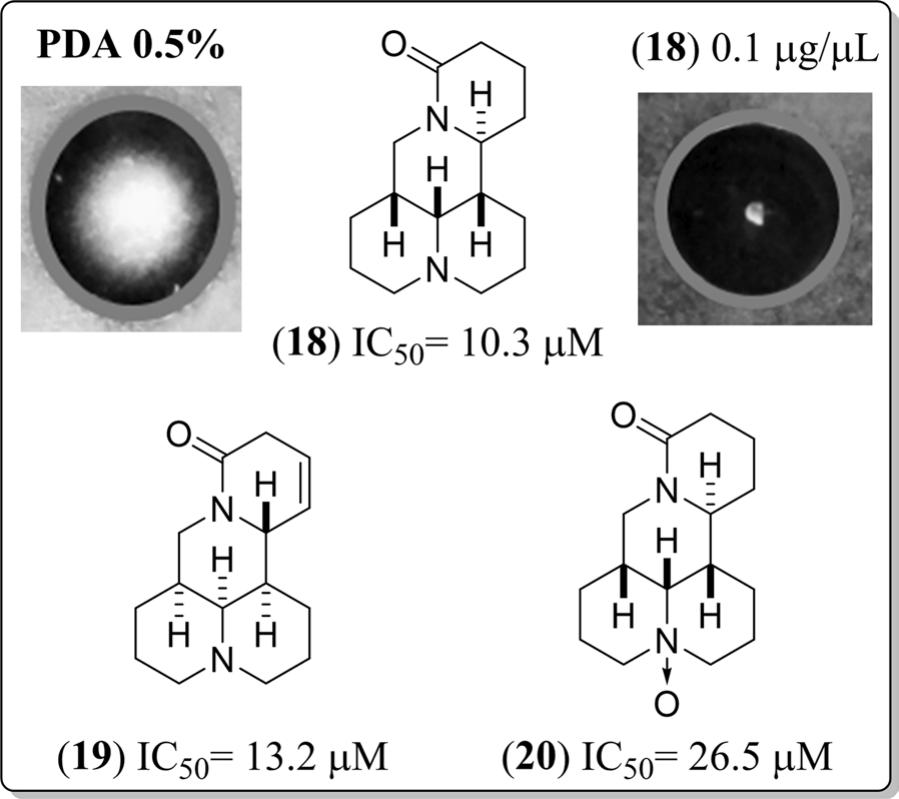
Matrine-type diazatetracyclic QAs and their relationship with *F. oxysporym* mycelial growth inhibition

Finally, a comparison of the structure and antifungal activity between **18** (group **I**) and **19** and **20** highlighted some structural differences (i.e., different relative configuration, an unsaturation, and a N → O group) to be considered relevant for antifungal activity (Fig. 4). Homologs of **18,** such as **19** (i.e., Δ^12(13)^**-**( +)-**18**) (IC_50_ = 13.2 µM, FS), did not have the same fungicidal effects as **18**, inferring that unsaturations and/or configuration variations at these positions cause a slight decrease in IC_50_ values and were also responsible for the fungistatic and non-fungicidal activity of **19**. In the case of compound **20**, there was a decrease of more than 50% inhibition compared to compound **18**. However, the IC_50_ value was located in group **II** despite being fungistatic.

## Conclusions

The present study obtained several QAs (n = 20) involving structural differences according to six alkaloid types (i.e., lupanine, sparteine, lupinine, cytisine, tetrahydrocytisine, and matrine-type). In addition, structural variability, substitution patterns, and double bonds were found to be relevant in understanding the outcome changes on *Fox* mycelial growth inhibition. QA **12** showed the best antifungal activity (IC_50_ < 12 µM), and although **13–15** were structurally related to **12** but differed by certain substitutions (methyl, formyl), the IC_50_ values were lower (> 15 µM), suggesting an important influence on the antifungal activity depending on the substitution patterns at N12 (e.g., EDG at N12 may be responsible for better inhibition against *Fox*, while EWG leads to lower inhibition). In the case of bridged tetracycles, substitutions or unsaturations on the A-ring positively influenced the QA bioactivity, while substitutions or double bonds on the D-ring caused a decrease in the phytopathogen inhibition. Finally, the small set of structural variants of fused tetracyclic QAs did not exhibit substantial activity variations compared to **18**, which showed the best antifungal activity for this QA type. All compounds reported in this study (**1**–**20**) were isolated from the species under study, tested against a *Fox* strain, and explored their structure–activity relationships for the first time. Our findings might be considered relevant baseline information to develop QA-based fungicides in further studies oriented to *Fox*’s management and control.

## Experimental

### Plant material

Fresh leaves of *Genista monspessulana*, *L. polyphyllus 'rusell'*, and *L. mutabilis* were used to isolated QAs. *Lupinus* and *Genista* plants were propagated from seeds under greenhouse conditions (vide infra). Seeds from *Lupinus* plants were commercially purchased from Sow Right Seeds [55]. Healthy seeds from *G. monspessulana* (voucher specimen No COL: 576,283 kept at Colombian National Herbarium) were collected from wild plants at Choconta municipality, Cundinamarca, Colombia (Temperature = 15 ± 3 °C, 2660 masl, 5°04′30 ″ N 73°43′05 ″ W). As described below, leaves were collected to proceed with the alkaloid extraction from the collected plant material.

### Propagation of Lupinus plants under greenhouse conditions

The seeds of the above-mentioned fabaceous plants were planted in 72-cell seedbeds containing a combined substrate of loamy-silty soil (LSS) with rice husk (RiH) at a 3:1 ratio. These plants were maintained under greenhouse conditions (temperature = 21 ± 4 °C; relative humidity (RH) = 65 ± 15%, altitude = 2562 masl, total light transmission = 85 ± 5%, total light diffusion = 55 ± 5%, and UV transmission between 290 and 340 nm = 5%) for 30 days. After 10–15 days, the seeds started the germination process. After 20–30 days of seed planting, the first and second pairs of leaves emerged, at which time the seedlings were transplanted into bags of 2 L capacity on a substrate containing a mixture of LSS/RiH 1:3 and were maintained under the same greenhouse conditions. After transplanting, the pot-planted seedlings were irrigated with water (500 mL) every 2 days, and once the elongation of the central axis began to be observed, commercial triple-15 fertilizer at a concentration of 5% was applied. After 70 ± 19 days of development under these conditions, plants reach the flowering stage. At this moment, fresh leaves were collected for QA extraction, as described below.

### Acid-based extraction of alkaloids

Fresh leaves (500 g) of each plant mentioned above were extracted using 0.5 M HCl (20 mL) under stirring at 130 rpm in an orbital shaker for 24 h. Subsequently, the acidic solution was filtered and brought to pH 10 with a 15% aqueous NH_3_ solution_._ Subsequently, liquid–liquid extraction was performed using chloroform to obtain an alkaloid-rich organic phase. Finally, the solvent excess was removed by distillation under reduced pressure at 375 mbar for 5 min, and the respective *Lupinus*- and *Genista*-derived quinolizidine-rich extracts (QREs) were obtained to be separated further by chromatographic procedures (vide infra).

### Thin-layer chromatography (TLC) and column chromatography (CC)

Thin-layer chromatography (TLC) was performed using Silica gel 60 F_254_. Different solvent mixtures by combining *n*-hexane (Hex), chloroform (CHCl_3_), dichloromethane (DCM), toluene (Tol), methanol (MeOH), even ammonia (NH_3_) and/or formic acid (HCOOH) reagents, were used as mobile phases. Dragendorff's reagent, iodine vapors, and UV at 254 and 366 nm were used as revealers to visualize analytes. Column chromatography (CC) involved gradient elution for separating and purifying target compounds. The stationary phase was silica for column chromatography (0.063–0.200 mm) (Merck), and different mixtures of the solvents mentioned above in different ratios were used as the mobile phases. TLC monitored CC separations.

### Isolation of quinolizidine alkaloids

Alkaloids **1**–**20** were isolated from *L. mutabilis*, *L. polyphyllus* '*rusell*', *G. monspessulana* following the procedures described below.

#### Isolation of alkaloids 1, 3, and 8

Fresh *L. mutabilis* leaves (200 g) were collected, ground, and extracted using the above-mentioned acid–base procedure (vide supra) to obtain the respective QRE (1.3234 g), monitored by TLC (DCM/MeOH/NH_3_ 8.5:1:0.5) and separated by CC (DCM/MeOH/NH_3_ 8.5:1:0.5). Fourteen fractions (Frs.) were gathered after TLC analysis (Frs. *Lm*1 to *Lm*14) and analyzed by GC–MS. Chromatographic analysis showed that Frs. *Lm*1 to *Lm*4 corresponded to pure **1** (217.2 mg), Frs. *Lm*5 to *Lm*7 were a **1**, **3** mixture, Frs. *Lm*8 to *Lm*11 involved a **1–2** mixture, and, finally, Frs. *Lm*12 ro *Lm*14 afforded the pure **8** (103.7 mg). Consecutively, the Frs. *Lm*5-to-*Lm*7 were separated by CC using gradient elution starting from the DCM/MeOH/HCOOH 9:1:1 mixture. This separation afforded five gathered fractions (Frs. *Lm*5-7a to *Lm*5-7e). In this sense, the Frs. *Lm*5-7a and *Lm*5-7b were the pure **1**, the Fr. *Lm*5-7c was a **1**, **3** mixture**,** and Frs. *Lm*5-7d and Fr. *Lm*5-7e were the pure **3** (13.5 mg).

#### Isolation of alkaloids 2, 5, 7, 11, 16–18, and 20

Fresh *L. polyphyllus ‘rusell’* leaves (300 g) were collected, ground, and extracted by the acid–base procedure to obtain the respective QRE (756.4 mg). A portion of this QRE (500 mg) was employed and separated by CC, using gradient elution from a DCM/MeOH/NH_3_ 8:1:1 mixture. The chromatographic separation afforded eight gathered fractions (*Lp*1 to *Lp*8). The depuration and purification by CC of fractions *Lp*2 and *Lp*4 yielded compounds **2** (9.8 mg) and **7** (15.6 mg), respectively. A new portion of fresh leaves was subsequently obtained after a sixth pruning event of the propagated *L. polyphyllus ‘rusell’*. These leaves (330 g) were subjected to an acid–base extraction process to obtain another QRE (1254.4 mg). A portion of this QRE (500 mg) was separated by CC using gradient elution from a DCM/MeOH/NH_3_ 8:1:1. Eighteen gathered fractions were obtained (Frs. *Lp*6-1 to *Lp*6-18). Frs. *Lp*6-1-to-*Lp6*-3 were a **1**, **11** mixture, Fr. *Lp*6-4 was the pure **11** (10.1 mg), Frs. *Lp*6-5-to-*Lp6-*7 were the pure **17** (32.5 mg), Frs. *Lp*6-8-to-*Lp*6-10 were a **5**, **16** mixture, Frs. *Lp*6-11-to-*Lp*6-14 were a **5, 16, 18, 19** mixture, and finally, Frs. *Lr*6-15-to-*Lp*6-18, were a **5, 18, 19** mixture**.** A further separation by CC for Frs. *Lp*6-8-to-*Lp*6-10 was carried out with isocratic elution (DCM/MeOH/HCOOH 8:1:1) and afforded six gathered fractions (Frs. *Lp*6-8-10a to *Lp*6-8-10f). Fr. *Lp*6-8-10a was the pure **16** (55.8 mg), Frs. *Lp*6-8-10b-to-*Lp*6-8-10e were a **5, 16** mixture**,** and Fr. *Lp*6-8-10f was the pure **5** (23.6 mg). Similarly, a further CC separation of the Fr. *Lp*6-15-to-*Lp*6-18 was performed using a gradient elution starting from a DCM/MeOH/HCOOH 7:2:1 mixture. This separation afforded five gathered fractions (*Lp*6-15-18a to *Lp*6-15-18e). Fr. *Lp*6-15-18a was the pure **18** (15.6 mg), Frs. *Lpr*6-15–18(b-c) were an **18, 19** mixture, and Frs. *Lpr*6-15-18d-e were the pure **19** (16.3 mg).

#### Isolation of alkaloids 9, 10, 12–15, and 19

Fresh leaves (500 g) of *G. monspessulana* were used to obtain the respective QRE (10.2132 g). A CC separation was performed using gradient elution starting from a DCM/MeOH/NH_3_ 8:1:1 mixture and affording ten gathered fractions (Frs. *Gm*1 to *Gm*10). The resulting fractions were additionally purified by successive CC (gradient elution) to obtain the compounds **9** (1407.4 mg, Fr. *Gm*2), **10** (29.3 mg, Fr. *Gm*4), **12** (264.2 mg, Fr. *Gm*7), **13** (23 mg, Fr. *Gm*3), **14** (14.2 mg, Fr. *Gm*3), **15** (32.1 mg, Fr. *Gm*6), and **19** (16.3 mg, Fr. *Gm*6), using DCM/MeOH/NH_3_ mixtures (7.0:2.5:0.5, 8.0:1.5:0.5, and 9.0:0.5:0.5).

### Gas chromatography coupled with mass spectrometry (GC-MS)

The GC–MS analyses were obtained with a Thermo Trace 1300 coupled to an ISQ LT mass spectrometer with a single quadrupole analyzer. For the analysis, a Rxi® 5Sil MS column with 60 m, 0.25 mm ID, and 0.25 μm (5% diphenyl/95% dimethylpolysiloxane) was used. A temperature program was implemented. The starting temperature was 120 °C, which was maintained for 2 min, and then a 6 °C/min ramp was applied until 300 °C was reached, which was maintained for 10 min. The injection volume was 1 μL in *split* mode, with a flow of 1 mL/min and a *split* ratio of 30. The transfer line temperature was 250 °C, and the carrier gas was grade-5 helium. The ionization mode was electron impact (EI) at 70 eV. The analyzed extracts were prepared at 1 µg/µL in CH_2_Cl_2_ (GC–MS-SupraSolv® grade). Retention indices were calculated using a series of C_10_-C_24_ *n*-alkanes from identical GC–MS analysis [56].

### High-performance liquid chromatography coupled with mass spectrometry analysis

High-resolution mass spectrometry analyses with electrospray ionization (ESI) were performed using a Bruker micrOTOF–QII mass spectrometer coupled to a Shimadzu Prominence liquid chromatography system, consisting of two analytical pumps model LC-20AD, with SIL-20AHT automatic injector, SPD-20A UV/Vis detector, CTO-20A column oven, CBM-20A controller. Each compound analyzed by this technique was prepared at 1 mg/mL using LCMS-grade methanol. The column used was a Phenomenex Luna C18 (5 µm, 150 × 2 mm). The flow was 0.2 mL/min, and the mobile phase was a mixture of solvents A (0.1% HCOOH in H_2_O) and B (0.1% formic acid in MeOH). The gradient started at 5% B (0 min) and was maintained for 2 min. Then, B was incremented to 100% from 5 to 30 min and maintained for 5 min. The oven temperature was 40 °C, and the wavelength was 254 and 280 nm. The ESI interface was operated in positive ion mode with 4.5 kV in the capillary and 0.5 kV in the endplate offset. The pressure of the nebulization gas was 0.4 Bar; the drying gas was maintained at a flow rate of 8 L/min at 200 °C. The collision and the quadrupole energy were set to 12 and 6 eV, respectively. RF1 and RF2 funnels were programmed to 150 and 200 Vpp, respectively. The mass spectra were calibrated using sodium formate.

### Nuclear magnetic resonance analysis

The ^1^H NMR and ^13^C NMR spectra (500 and 125 MHz, respectively) were recorded on a DRX 500 spectrometer (Bruker) using CDCl_3_ or CD_3_OD as a solvent with tetramethylsilane (TMS, 0.05% v/v) as an internal standard at room temperature. Chemical shifts are indicated in δ (ppm) regarding TMS, and coupling constants are expressed in Hertz (Hz). Each spectrum resulted from 128 scans with pulse widths (PW) of 8.0 µs (30 °C) and relaxation delays (RD) of 6.0 s.

### Specific rotation

The specific rotation data of the QAs **1–20** were recorded by using Jasco P-2000 polarimeter. Spectrophotometric grade methanol was used as a solvent, and the values were reported in terms of specific rotation (${[\alpha ]}_{D}^{20}$).

### Antifungal activity against *Fox* of QAs 1-20

The studied phytopathogen was a virulent isolate (*Fox* LQB-03) obtained from wilting *Physalis peruviana* plants [57]. This isolate was reactivated in potato dextrose agar (PDA) to be used in the antifungal assays. The in vitro antifungal activity evaluation of QAs was performed by measuring the mycelial radial growth of the phytopathogen *Fox* in the presence of the test compounds at different concentrations compared to that of a blank (PDA 0.5%), using a 12-well plate amended semi-solid medium assay [58]. The culture medium containing 2.4% PDB and 1.5% bacteriological agar in 100 mL of distilled water was initially prepared. The medium was homogenized for 2 min in a microwave oven and then sterilized in an autoclave for 1 h at 120 °C. Medium (20 mL) was placed in a previously sterilized Petri dish, and once it cooled and solidified, a 2-mm plug from a previously prepared monosporic culture was placed in the central part of the Petri dish and left to grow at 28 °C for 8 days to propagate the fungus.

Several concentrations of each test compound (0.1–1000 µg/mL) were prepared using serial dilutions by dispersing them in 0.5% PDA medium for the antifungal assays. Subsequently, each concentration of every QA was considered a treatment, and they were randomly placed in a 12-well glass plate (size 79 × 63 × 4 mm). Finally, a 1-mm plug from an 8-day fungal monosporic culture, having a diameter proportional to a borosilicate capillary tube of 32 mm, was taken and placed in the central part of each well containing a treatment. This plate was placed in a humid chamber for 72 h at 25 °C. After incubation, a photograph of the 12-well glass plate was recorded and analyzed in ImageJ software. The growth area was measured, and the inhibition percentage was then calculated. The IC_50_ was obtained from dose–response curves (i.e., inhibition percentages *versus* the decadic logarithm of the concentration) by nonlinear regression in GraphPad Prism 5.0 software. The evaluation of each concentration of each treatment was performed in triplicate.

### Fungicidal (FC) and fungistatic (FS) activity

For the FC or FS activity classification procedure, the plug of the phytopathogen used in the prior treatments was taken at 1 µg/µL and was placed on a fresh, unsupplemented PDA medium for 72 h. After this time, mycelial growth was also observed. In the case of further mycelial growth, the QA was classified as fungistatic (FS), and in the case of no mycelial growth, the QA was classified as fungicidal (FC) [59].

### Data analysis

A Shapiro-Wilks normality test was initially carried out to examine the normal distribution of the data (*p* > 0.05). Once the normal data distribution was verified, an analysis of variance (ANOVA) was then accomplished, followed by a posthoc Tukey test to establish significant differences between treatments (*p* < 0.05). These analyses were performed in Infostat statistical software [60].

## Supplementary Information

**Additional file 1**: **Figure S1**. Chemical structures of isolated QAs **1–20**, grouped according to QA types. **Figure S2**. a). GC–MS of **1**; b). EIMS of **1**; c). HRESIMS of **1**. **Figure S3.** a). GC–MS of **2**; b). EIMS of **2**; c). HRESIMS of **2**. **Figure S4**. a). GC–MS of **3**; b). EIMS of **3**; c). HRESIMS of **3**. **Figure S5**. a). GC–MS of **4**; b). EIMS of **4**; c). HRESIMS of **4**. **Figure S6**. a). GC–MS of **5**; b). EIMS of **5**; c). HRESIMS of **5**. **Figure S7**. a). GC–MS of **6**; b). EIMS of **6**; c). HRESIMS of **6**. **Figure S8**. a). GC–MS of **7**; b). EIMS of **7**; c). HRESIMS of **7**. **Figure S9**. a). GC–MS of **8**; b). EIMS of **8**; c). HRESIMS of **8**. **Figure S10**. a). GC–MS of **9**; b). EIMS of **9**; c). HRESIMS of **9**. **Figure S11**. a). GC–MS of **10**; b). EIMS of **10**; c). HRESIMS of **10**. **Figure S12**. a). GC–MS of **11**; b). EIMS of **11**; c). HRESIMS of **11**. **Figure S13**. a). GC–MS of **12**; b). EIMS of **12**; c). HRESIMS of **12**. **Figure S14**. a). GC–MS of **13**; b). EIMS of **13**; c). HRESIMS of **13**. **Figure S15**. a). GC–MS of **14**; b). EIMS of **14**; c). HRESIMS of **14**. **Figure S16**. a). GC–MS of **15**; b). EIMS of **15**; c). HRESIMS of **15**. **Figure S17**. a). GC–MS of **16**; b). EIMS of **16**; c). HRESIMS of **16**. **Figure S18**. a). GC–MS of **17**; b). EIMS of **17**; c). HRESIMS of **17**. **Figure S19**. a). GC–MS of **18**; b). EIMS of **18**; c). HRESIMS of **18**. **Figure S20**. a). GC–MS of **19**; b). EIMS of **19**; c). HRESIMS of **19**. **Figure S21**. a). GC–MS of **20**; b). EIMS of **20**; c). HRESIMS of **20**.

## Data Availability

All data generated and analyzed during this study are included in this published article and its supplementary information file.
