# Supplementary material for: Antifungal activity against Fusarium oxysporum of quinolizidines isolated from three controlled-growth Genisteae plants: structure–activity relationship implications
Source: Nat Prod Bioprospect. 2023 Mar 20;13(1):9. doi: 10.1007/s13659-023-00373-4 (PMC10027967; doi:10.1007/s13659-023-00373-4)
Supplement: Supplementary file 1 — Additional file 1: Figure S1. Chemical structures of isolated QAs 1–20, grouped according to QA types. Figure S2. a). GC–MS of 1; b). EIMS of 1; c). HRESIMS of 1. Figure S3. a). GC–MS of 2; b). EIMS of 2; c). HRESIMS of 2. Figure S4. a). GC–MS of 3; b). EIMS of 3; c). HRESIMS of 3. Figure S5. a). GC–MS of 4; b). EIMS of 4; c). HRESIMS of 4. Figure S6. a). GC–MS of 5; b). EIMS of 5; c). HRESIMS of 5. Figure S7. a). GC–MS of 6; b). EIMS of 6; c). HRESIMS of 6. Figure S8. a). GC–MS of 7; b). EIMS of 7; c). HRESIMS of 7. Figure S9. a). GC–MS of 8; b). EIMS of 8; c). HRESIMS of 8. Figure S10. a). GC–MS of 9; b). EIMS of 9; c). HRESIMS of 9. Figure S11. a). GC–MS of 10; b). EIMS of 10; c). HRESIMS of 10. Figure S12. a). GC–MS of 11; b). EIMS of 11; c). HRESIMS of 11. Figure S13. a). GC–MS of 12; b). EIMS of 12; c). HRESIMS of 12. Figure S14. a). GC–MS of 13; b). EIMS of 13; c). HRESIMS of 13. Figure S15. a). GC–MS of 14; b). EIMS of 14; c). HRESIMS of 14. Figure S16. a). GC–MS of 15; b). EIMS of 15; c). HRESIMS of 15. Figure S17. a). GC–MS of 16; b). EIMS of 16; c). HRESIMS of 16. Figure S18. a). GC–MS of 17; b). EIMS of 17; c). HRESIMS of 17. Figure S19. a). GC–MS of 18; b). EIMS of 18; c). HRESIMS of 18. Figure S20. a). GC–MS of 19; b). EIMS of 19; c). HRESIMS of 19. Figure S21. a). GC–MS of 20; b). EIMS of 20; c). HRESIMS of 20. [file 13659_2023_373_MOESM1_ESM.pdf]

**Supplementary Material**

# Antifungal Activity against *Fusarium oxysporum* of Quinolizidines Isolated from Three Controlled-Growth Genisteae Plants: Structure-Activity Relationship Implications

Willy Cely-Veloza[a,*], Lydia Yamaguchi[b], Diego Quiroga[a], Massuo J. Kato[b], Ericsson Coy-Barrera[a,*]

[a] Bioorganic Chemistry Laboratory, Universidad Militar Nueva Granada, Cajicá 250247, Colombia.
[b] Laboratório de Quimica de Produtos Naturais, University of São Paulo, São Paulo 26077, Brazil.

## Content

**Page**

**Figure S1**. Chemical structures of isolated QAs **1-20**, grouped according QA types. 2
**Figure S2**. **a).** GC-MS of **1**; **b).** EIMS of **1**; **c).** HRESIMS of **1**. 3
**Figure S3**. **a).** GC-MS of **2**; **b).** EIMS of **2**; **c).** HRESIMS of **2**. 4
**Figure S4**. **a).** GC-MS of **3**; **b).** EIMS of **3**; **c).** HRESIMS of **3**. 5
**Figure S5**. **a).** GC-MS of **4**; **b).** EIMS of **4**; **c).** HRESIMS of **4**. 6
**Figure S6**. **a).** GC-MS of **5**; **b).** EIMS of **5**; **c).** HRESIMS of **5**. 7
**Figure S7**. **a).** GC-MS of **6**; **b).** EIMS of **6**; **c).** HRESIMS of **6**. 8
**Figure S8**. **a).** GC-MS of **7**; **b).** EIMS of **7**; **c).** HRESIMS of **7**. 9
**Figure S9**. **a).** GC-MS of **8**; **b).** EIMS of **8**; **c).** HRESIMS of **8**. 10
**Figure S10**. **a).** GC-MS of **9**; **b).** EIMS of **9**; **c).** HRESIMS of **9**. 11
**Figure S11**. **a).** GC-MS of **10**; **b).** EIMS of **10**; **c).** HRESIMS of **10**. 12
**Figure S12**. **a).** GC-MS of **11**; **b).** EIMS of **11**; **c).** HRESIMS of **11**. 13
**Figure S13**. **a).** GC-MS of **12**; **b).** EIMS of **12**; **c).** HRESIMS of **12**. 14
**Figure S14**. **a).** GC-MS of **13**; **b).** EIMS of **13**; **c).** HRESIMS of **13**. 15
**Figure S15**. **a).** GC-MS of **14**; **b).** EIMS of **14**; **c).** HRESIMS of **14**. 16
**Figure S16**. **a).** GC-MS of **15**; **b).** EIMS of **15**; **c).** HRESIMS of **15**. 17
**Figure S17**. **a).** GC-MS of **16**; **b).** EIMS of **16**; **c).** HRESIMS of **16**. 18
**Figure S18**. **a).** GC-MS of **17**; **b).** EIMS of **17**; **c).** HRESIMS of **17**. 19
**Figure S19**. **a).** GC-MS of **18**; **b).** EIMS of **18**; **c).** HRESIMS of **18**. 20
**Figure S20**. **a).** GC-MS of **19**; **b).** EIMS of **19**; **c).** HRESIMS of **19**. 21
**Figure S21**. **a).** GC-MS of **20**; **b).** EIMS of **20**; **c).** HRESIMS of **20**. 22

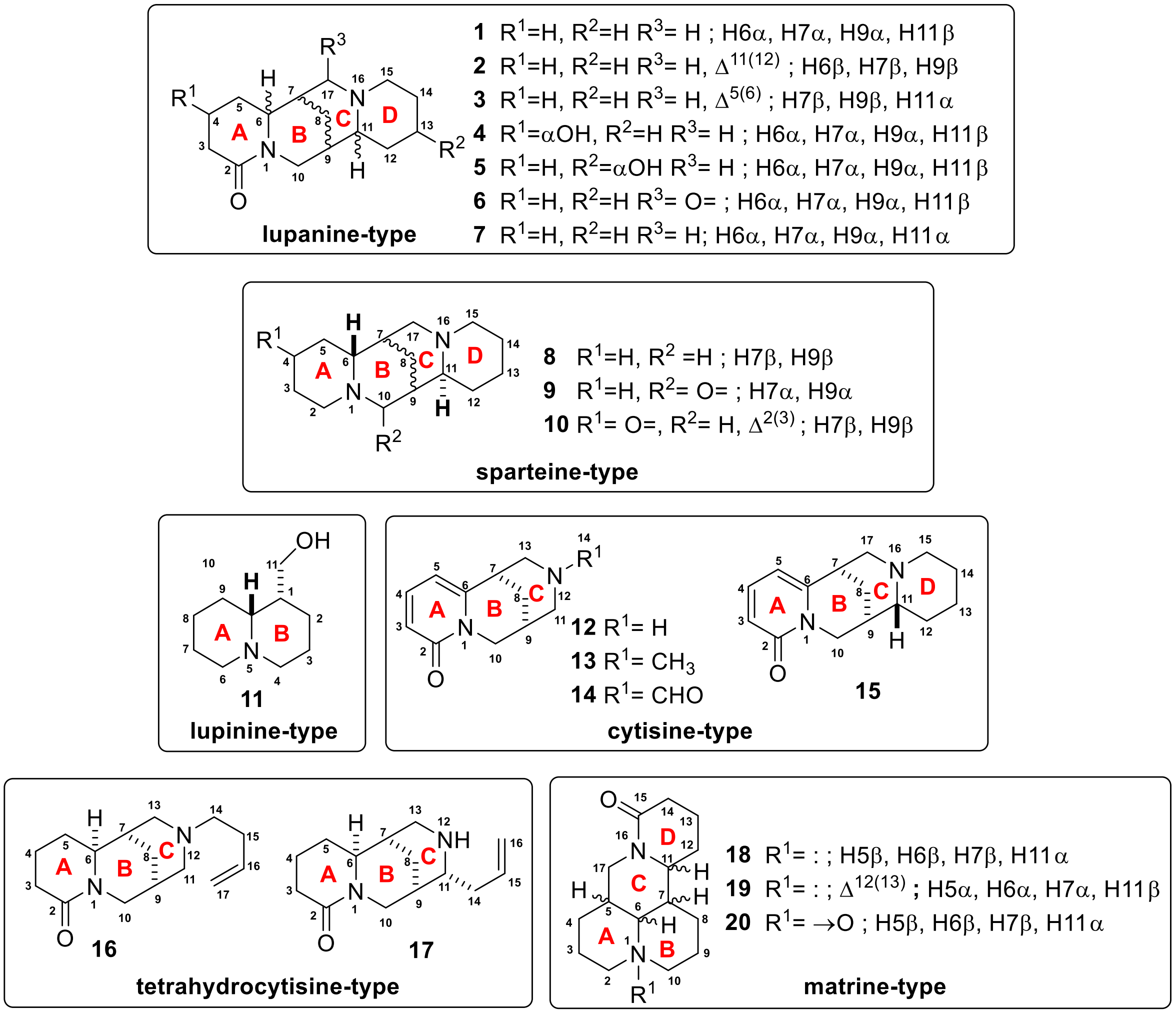

**Figure S1**. Chemical structures of isolated QAs **1-20**, grouped according QA types.

## 1. <u>Compounds 1-7: Lupanine-type</u>

Compounds **1-7** were isolated from the leaves of *L. polyphyllus 'rusell'* and *L. mutabilis*. Each lupanine-type QA was structurally characterized by the presence of a bridged tetracycle with a lactam group in the A ring. Compounds **2** and **3** were structurally related to compound **1**, but these differ by the presence of a double bond in D-ring (in **2)** and in A-ring (in **3)**. GC-MS and HRMS analyses of **2** and **3** showed the same molecular formula $C_{15}H_{22}N_2O$, indicating an isomeric relationship. To establish the difference between isomers **2** and **3**, $^1$H NMR was then used.

### 1.1. Compound 1: (–)-lupanine

Compound **1** ($[\alpha]_D^{20}$ = –70, MeOH, *c* 0.01) was a white crystalline solid (217.2 mg, mp. 98-100 °C), positive for Dragendorff's reagent, soluble in chloroform, methanol, and water. The analysis by GC-MS afforded an *m/z* = 248 that corresponds to the molecular formula $C_{15}H_{24}N_2O$. This information was confirmed by HRESIMS, and a $[M+H]^+$ = 249.1954 (calcd. 249.1966) was obtained. The structure of compound **1** was confirmed by $^1$H and $^{13}$C NMR, whose signals were compared with the literature and agreed with the data reported for (–)-lupanine, isolated from *Lupinus albus* [1].

**$^1$H NMR** (500 MHz, $CD_3OD$) $\delta_H$ 4.48 (d, *J* = 13.5 Hz, 1H), 3.43 (dd, *J* = 71.7, 11.3 Hz, 1H), 2.90 (d, *J* = 66.1 Hz, 1H), 2.68 (d, *J* = 13.7 Hz, 2H), 2.48 (ddd, *J* = 17.9, 13.3, 5.2 Hz, 2H), 2.42 – 2.36 (m, 2H), 2.33 – 2.20 (m,

2H), 2.02 – .89 (m, 2H), 1.87 – 1.83 (m, 6H), 1.72 – 1.62 (m, 2H), 1.58 – 1.52 (m, 3H). **$^{13}$C NMR** (125 MHz, $CD_3OD$) $\delta_C$ 171.9, 64.5, 60.9, 55.6, 52.4, 46.6, 34.4, 33.0, 32.8, 31.8, 27.4, 26.5, 24.4, 23.9, 19.5.

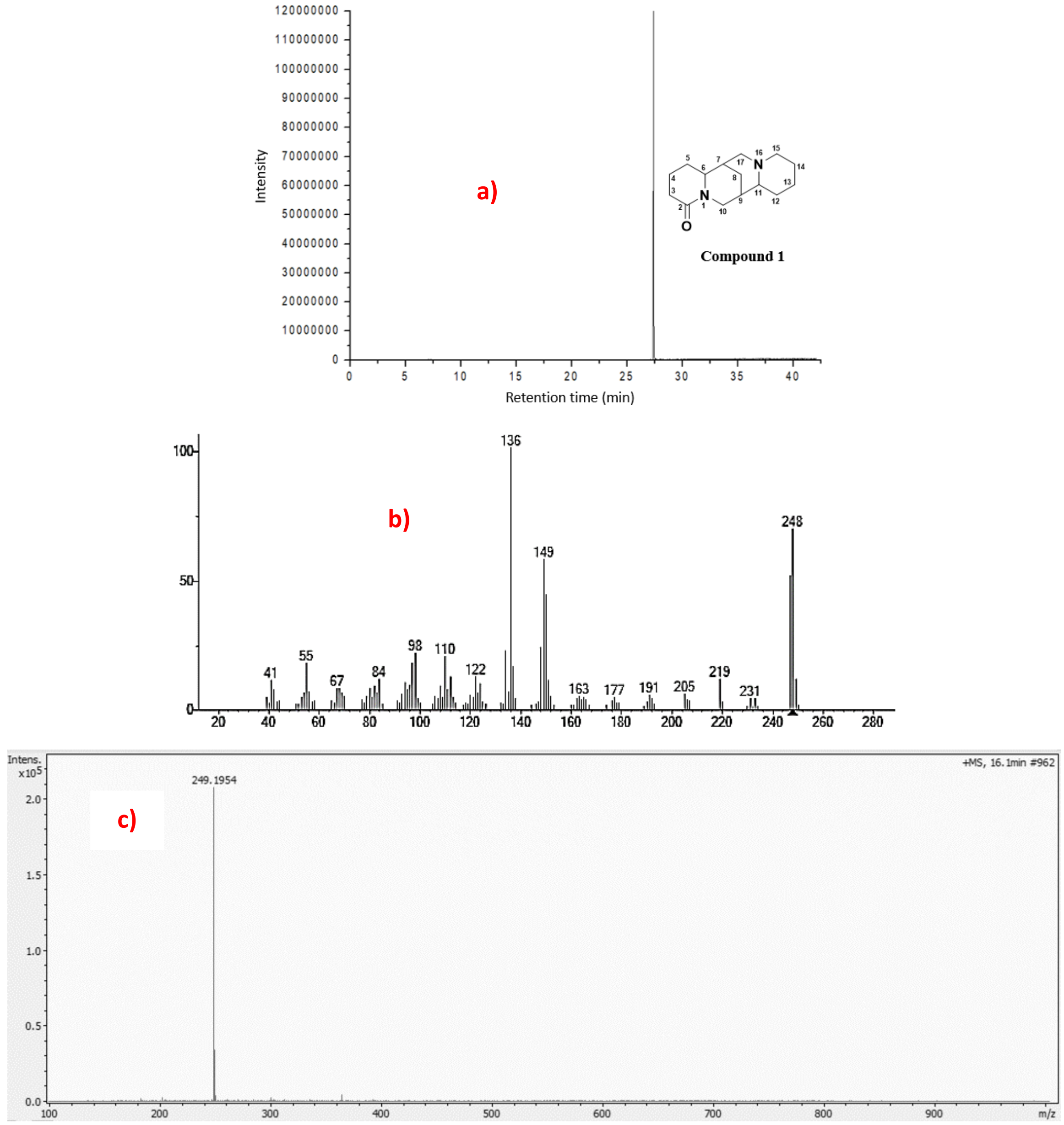


**Figure S2**. a). Chromatographic profile obtained by GC-MS of **1**; b). Mass spectra by electron impact at 70eV of **1**; c). Mass spectrum by HRESIMS of **1**.

## 1.2. Compound 2: (+)-11,12-dehydrolupanine

Compound **2** ($[\alpha]_D^{20}$ = +72, MeOH, *c* 0.02) was an oily substance (9.8 mg), positive for Dragendorff's reagent, soluble in chloroform, methanol, and water. The analysis by GC-MS afforded an *m/z* = 246 that corresponds to the molecular formula $C_{15}H_{22}N_2O$. This information was confirmed by HRESIMS, and a $[M+H]^+$ = 247.1813 (calcd. 247.1810) was obtained. The structure of compound **2** was confirmed by $^1$H and $^{13}$C NMR, whose signals were compared with the literature and agreed with the data reported for (+)-11,12-dehydrolupanine, isolated from *Thermopsis rhombifolia* [2].

**$^1$H NMR** (500 MHz, $CD_3OD$) $\delta_H$ 4.35 (d, J = 13.5 Hz, 1H), 4.17 (dd, J = 10.9, 5.6 Hz, 1H), 3.48 (dd, J = 10.0, 4.9 Hz, 1H), 3.41 – 3.34 (m, 2H), 3.22 (d, J = 11.7 Hz, 2H), 2.74 (d, J = 13.0 Hz, 1H), 2.23 (d, J = 11.7 Hz, 2H), 2.19 – 2.15 (m, J = 8.7, 4.6 Hz, 1H), 1.90 (d, J = 10.8 Hz, 2H), 1.81 (d, J = 3.9 Hz, 2H), 1.76 – 1.75 (m, 1H), 1.73 – 1.69 (m, 2H), 1.59 (d, J = 13.5 Hz, 2H), 1.54 – 1.43 (m, J = 16.6, 10.2, 6.2 Hz, 2H).

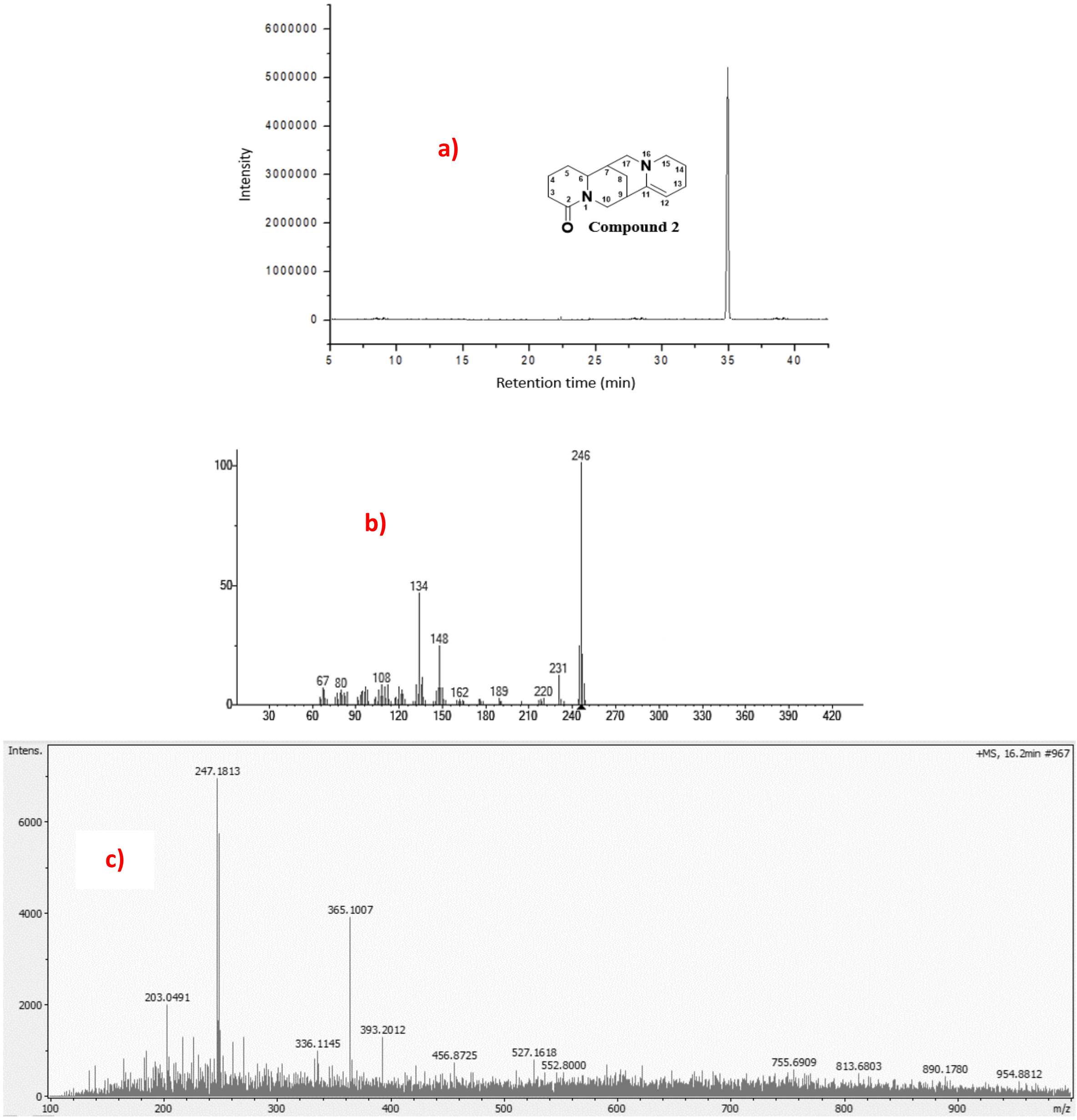


**Figure S3. a**). Chromatographic profile obtained by GC-MS of **2**; **b**). Mass spectra by electron impact at 70eV of **2**; **c**). Mass spectrum by HRESIMS of **2**.

## 1.3. Compound 3: (+)-5,6-dehydrolupanine

Compound **3** ($[\alpha]_D^{20}$ = +42, MeOH, *c* 0.02) was a yellow oily substance (13.3 mg), positive for Dragendorff's reagent, soluble in chloroform, methanol, and water. The analysis by GC-MS afforded an *m/z* = 246 that corresponds to the molecular formula $C_{15}H_{22}N_2O$. This information was confirmed by HRESIMS, and a $[M+H]^+$ = 247.1811 (calcd 247.1810) was obtained. The structure of compound **3** was confirmed by $^1$H and $^{13}$C NMR, whose signals were compared with the literature and agreed with the data reported for (+)-5,6-dehydrolupanine, isolated from *Anagyrus foetida* [3].

**$^1$H NMR** (500 MHz, $CD_3OD$) $\delta_H$ 5.22 (d, *J* = 0.6 Hz, 1H), 3.63 (d, *J* = 9.1 Hz, 1H), 3.12 (dd, *J* = 36.8, 20.5 Hz, 2H), 2.91 – 2.82 (m, *J* = 0.8 Hz, 2H), 2.81 – 2.70 (m, 2H), 2.45 – 2.35 (m, *J* = 17.1 Hz, 2H), 2.19 – 2.13 (m, *J* = 14.2 Hz, 2H), 2.06 – 2.00 (m, *J* = 9.7 Hz, 1H), 1.91 (d, *J* = 11.5 Hz, 1H), 1.88 – 1.85 (m, *J* = 5.4 Hz, 2H), 1.84 – 1.80 (m, *J* = 3.4 Hz, 1H), 1.79 – 1.73 (m, *J* = 12.3 Hz, 1H), 1.65 – 1.59 (m, 1H), 1.58 – 1.53 (m, 1H), 1.32 – 1.24 (m, 2H). **$^{13}$C NMR** (125 MHz, $CD_3OD$) $\delta_C$ 170.9, 142.9, 102.4, 63.3, 56.5, 54.8, 48.0, 34.0, 33.1, 31.8, 27.5, 25.0, 22.8, 21.5, 19.2.

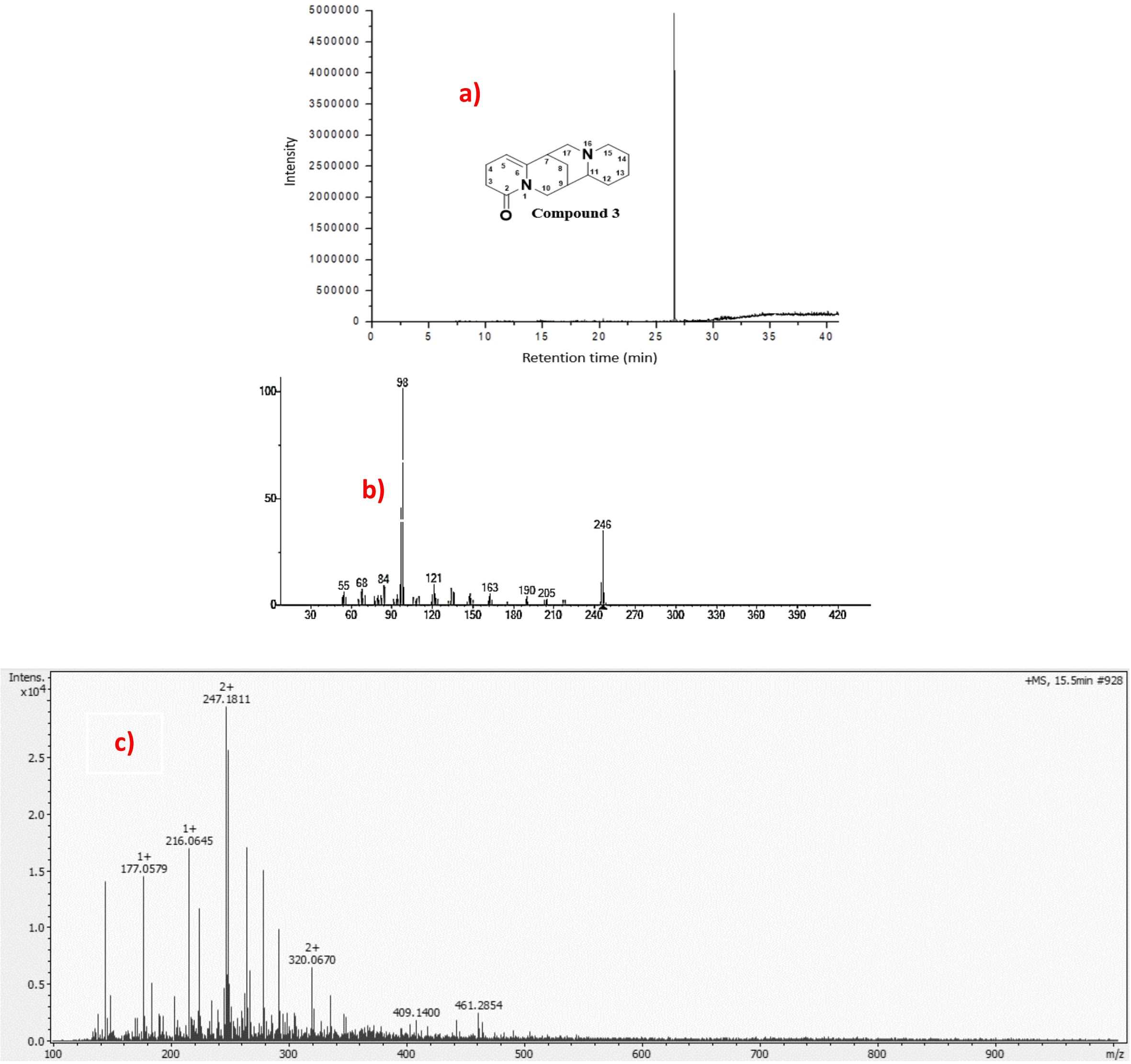


**Figure S4. a**) Chromatographic profile obtained by GC-MS of **3**; **b**). Mass spectra by electron impact at 70eV of **3**; **c**). Mass spectra by HRESIMS of **3**.

## 1.4. Compound 4: (–)-4α-hydroxylupanine

Compound **4** ($[\alpha]_D^{20}$ = –35, MeOH, *c* 0.01) was a purple oil (116.6 mg), positive for Dragendorff's reagent, and soluble in methanol and water. The analysis by GC-MS afforded an *m/z* = 264 that corresponds to the molecular formula $C_{15}H_{24}N_2O_2$. This information was confirmed by HRESIMS, and a $[M+H]^+$ = 265.1924 (calcd 265.1916) was obtained. The structure of compound **4** was confirmed by $^1H$ and $^{13}C$ NMR, whose signals were compared with the literature and agreed with the data reported for (–)-4α-hydroxylupanine [4].

**$^1$H NMR** (500 MHz, $CD_3OD$) $\delta_H$ 4.17 (dd, *J* = 10.9, 5.6 Hz, 1H), 3.48 (dd, *J* = 10.0, 4.9 Hz, 1H), 3.41 – 3.34 (m, 1H), 3.22 (d, *J* = 11.7 Hz, 1H), 2.74 (d, *J* = 13.0 Hz, 2H), 2.63 (d, *J* = 10.2 Hz, 1H), 2.44 (d, *J* = 10.7 Hz, 1H), 2.27 – 2.12 (m, 4H), 1.90 (d, *J* = 10.8 Hz, 2H), 1.84 – 1.78 (m, 3H), 1.77 – 1.70 (m, 4H), 1.59 (d, *J* = 13.5 Hz, 1H), 1.54 – 1.42 (m, 1H). **$^{13}$C NMR** (125 MHz, $CD_3OD$) $\delta_C$ 174.0, 77.2, 68.2, 64.4, 61.8, 55.5, 52.7, 47.9, 34.5, 33.2, 32.2, 27.3, 26.4, 24.9, 24.5, 24.5.

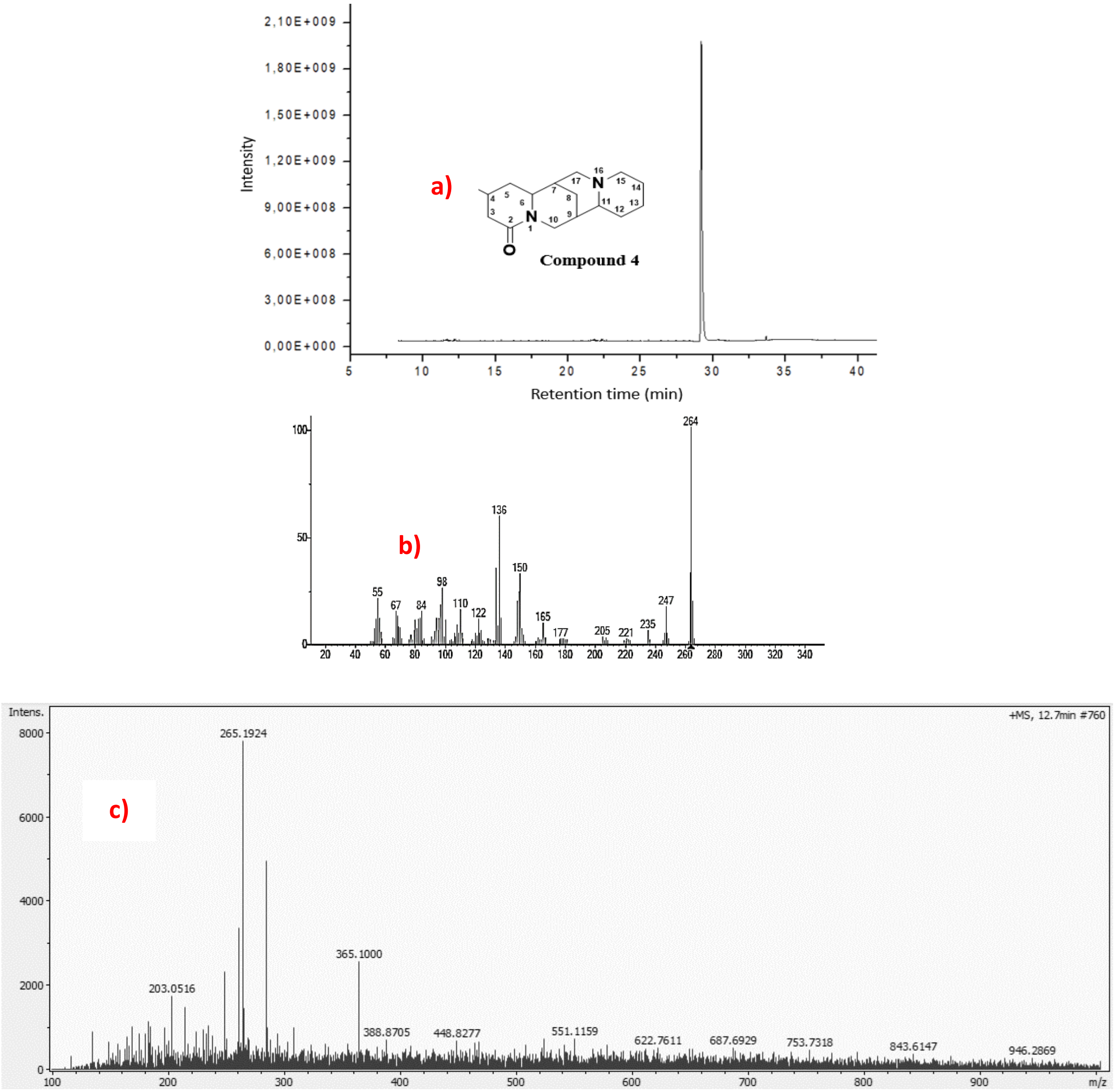


**Figure S5. a**). Chromatographic profile obtained by GC-MS of **4**; **b**). Mass spectra by electron impact at 70eV of **4**; **c**). Mass spectrum by HRESIMS of **4**.

## 1.5. Compound 5: (–)-13α-hydroxylupanine

Compound **5** ($[\alpha]_D^{20}$ = –82, MeOH, *c* 0.01) was a brown solid (23.6 mg, mp.170-173ºC), positive for Dragendorff's reagent, soluble in chloroform, methanol, and water. The analysis by GC-MS afforded an *m/z* = 264 that corresponds to the molecular formula $C_{15}H_{24}N_2O_2$. This information was confirmed by HRESIMS, and a $[M+H]^+$ = 265.1910 (calcd 265.1916) was obtained. The structure of compound **5** was confirmed by $^1H$ and $^{13}C$ NMR, whose signals were compared with the literature and agreed with the data reported for (–)-13α-hydroxylupanine [5].

**$^1$H NMR** (500 MHz, $CDCl_3$) $\delta_H$ 4.47 (dt, *J* = 13.2, 2.4 Hz, 1H), 4.08 – 4.04 (m, 1H), 3.30 (dd, *J* = 10.0, 5.0 Hz, 1H), 2.91 – 2.85 (m, 1H), 2.56 – 2.54 (m, 1H), 2.50 (dd, *J* = 13.3, 2.4 Hz, 1H), 2.47 – 2.41 (m, 1H), 2.38 (dd, *J* = 12.6, 2.2 Hz, 1H), 2.34 (dd, *J* = 8.4, 4.1 Hz, 1H), 2.32 – 2.26 (m, 1H), 2.21 – 2.15 (m, 1H), 2.13 – 2.09 (m, 1H), 2.09 – 2.04 (m, 1H), 2.01 – 1.96 (m, 1H), 1.84 – 1.82 (m, 1H), 1.81 – 1.79 (m, 1H), 1.78 – 1.74 (m, 1H), 1.68 – 1.65 (m, 2H), 1.63 – 1.62 (m, 1H), 1.61 – 1.58 (m, 1H), 1.57 – 1.54 (m, 1H), 1.51 (dd, *J* = 13.5, 3.1 Hz, 1H), 1.27 (dt, *J* = 12.4, 2.3 Hz, 1H). **$^{13}$C NMR** (125 MHz, $CDCl_3$) $\delta_C$ 171.9, 64.4, 60.9, 57.2, 52.5, 49.4, 46.8, 40.1, 34.3, 33.1, 32.3, 31.7, 27.4, 26.6, 19.7.

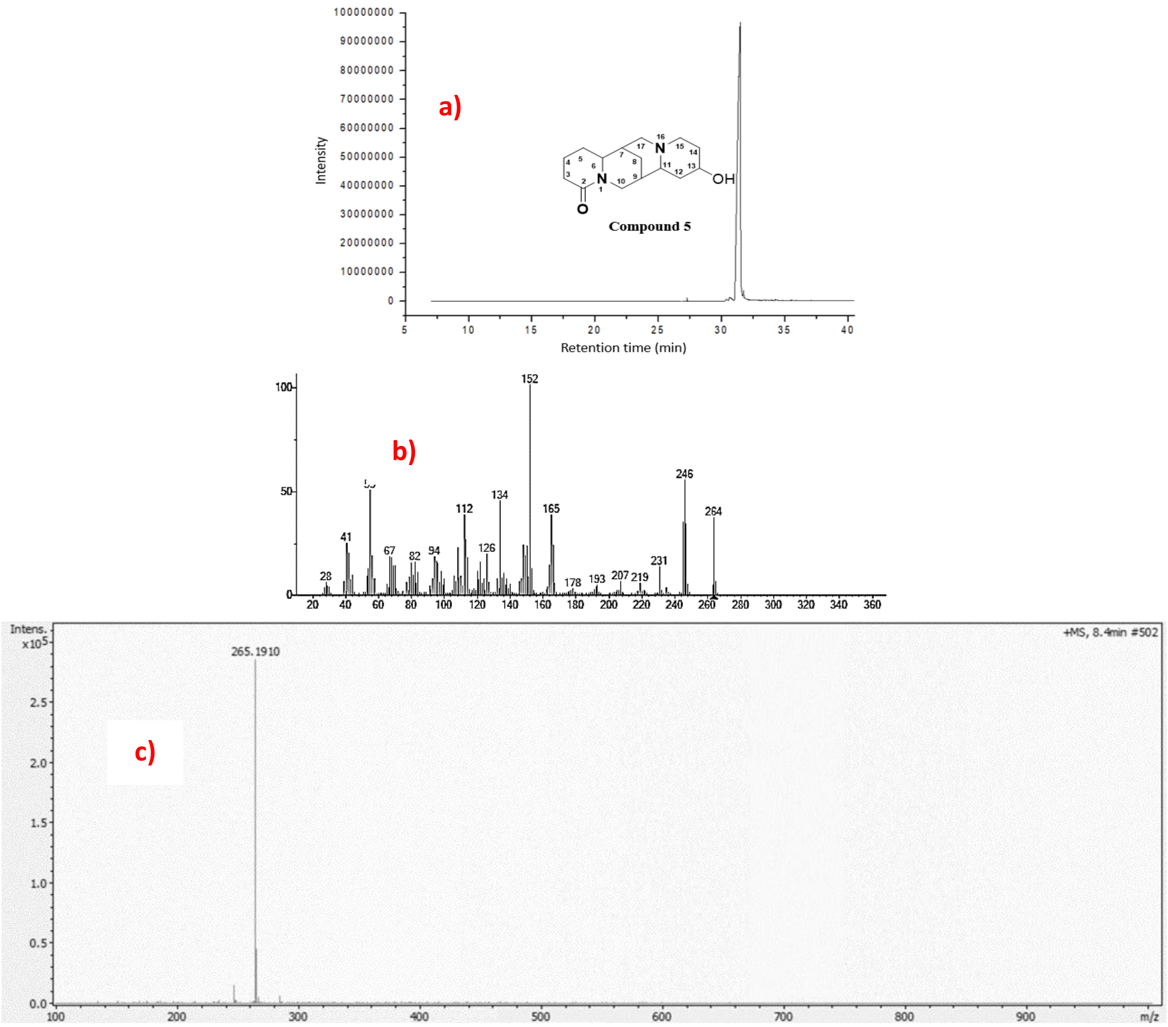


**Figure S6. a**). Chromatographic profile obtained by GC-MS of **5**; **b**). Mass spectra by electron impact at 70eV of **5**; **c**). Mass spectrum by HRESIMS of **5**.

### 1.6. Compound 6: (–)-17-oxolupanine

Compound **6** ($[\alpha]_D^{20}$ = –40, MeOH, *c* 0.02) was a yellow oil (7.9 mg), positive for Dragendorff's reagent, soluble in chloroform, methanol, and water. The analysis by GC-MS afforded an *m/z* = 262 that corresponds to the molecular formula $C_{15}H_{22}N_2O_2$. This information was confirmed by HRESIMS, and a $[M+H]^+$ = 263.1748 (calcd 263.1759) was obtained. The structure of compound **6** was confirmed by $^1H$ and $^{13}C$ NMR, whose signals were compared with the literature and agreed with the data reported for (–)-17-oxolupanine [6].

**$^1$H NMR** (500 MHz, $CDCl_3$) $\delta_H$ 3.91 (dd, *J* = 21.0, 10.9 Hz, 1H), 3.68 – 3.63 (m, 2H), 3.48 (d, *J* = 6.2 Hz, 1H), 3.07 (dd, *J* = 11.8, 3.6 Hz, 1H), 2.95 (d, *J* = 11.9 Hz, 1H), 2.72 (dd, *J* = 9.8, 2.4 Hz, 1H), 2.54 – 2.50 (m, 1H), 2.35 (dt, *J* = 4.7, 2.3 Hz, 1H), 2.02 (d, *J* = 3.6 Hz, 1H), 2.00 – 1.95 (m, 1H), 1.91 – 1.87 (m, 2H), 1.85 – 1.81 (m, 2H), 1.79 (d, *J* = 3.8 Hz, 1H), 1.77 (d, *J* = 4.1 Hz, 1H), 1.75 (d, *J* = 4.0 Hz, 1H), 1.60 – 1.59 (m, 2H), 1.58 (d, *J* = 2.4 Hz, 1H), 1.57 (d, *J* = 3.6 Hz, 1H). **$^{13}$C NMR** (125 MHz, $CDCl_3$) $\delta_C$ 170.7, 167.3, 61.1, 58.9, 48.0, 43.6, 42.9, 33.8, 33.4, 33.4, 32.8, 27.0, 25.3, 25.0, 19.4.

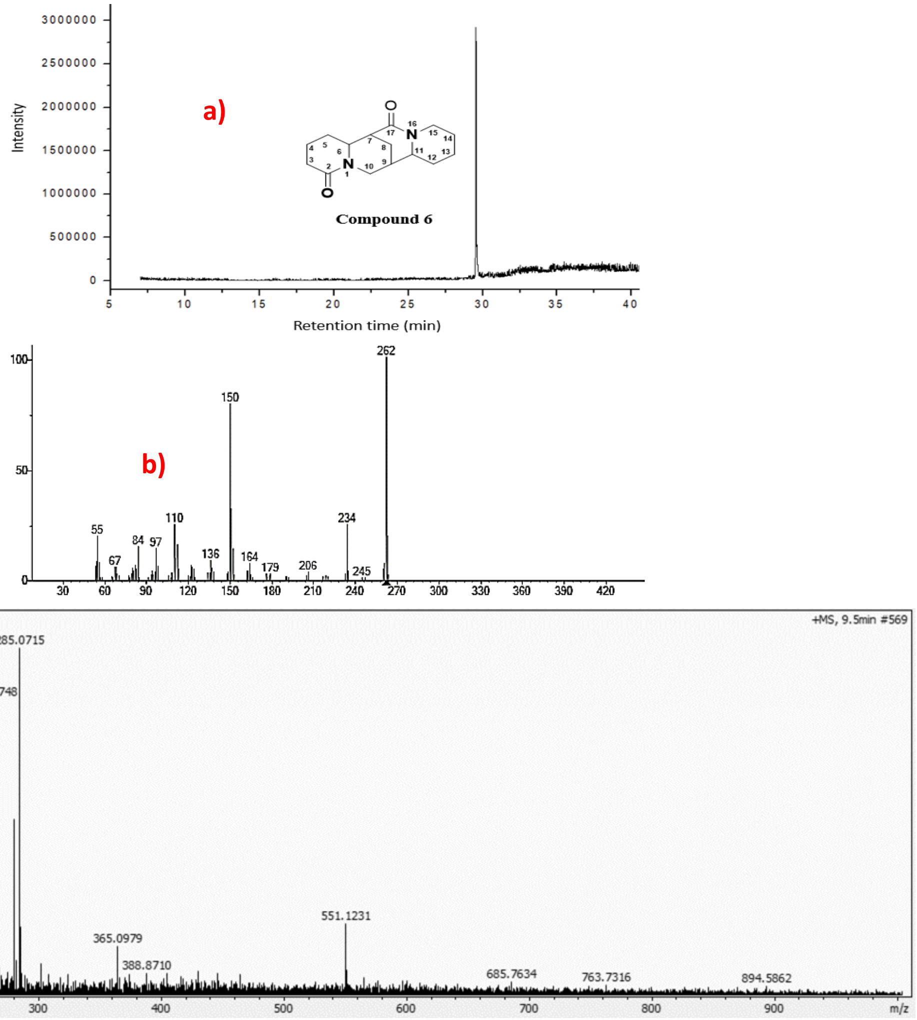


**Figure S7**. **a**). Chromatographic profile obtained by GC-MS of **6**; **b**). Mass spectra by electron impact at 70eV of **6**; **c**). Mass spectrum by HRESIMS of **6**.

### 1.7. Compound 7: (–)-α-isolupanine

Compound **7** ($[\alpha]_D^{20}$ = –20, MeOH, *c* 0.02) was a white crystalline solid (15.6 mg, mp. 99-100 °C), positive for Dragendorff's reagent, soluble in chloroform, methanol, and water. The analysis by GC-MS afforded an *m/z* = 248 that corresponds to the molecular formula $C_{15}H_{24}N_2O$. This information was confirmed by HRESIMS, and a $[M+H]^+$ = 249.1957 (calcd 249.1966) was obtained. The structure of compound **7** was confirmed by $^1H$ and $^{13}C$ NMR, whose signals were compared with the literature and agreed with the data reported for (–)-α-isolupanine, [7,8].

**$^1$H NMR** (500 MHz, $CD_3OD$) $\delta_H$ 4.55 (d, *J* = 13.5 Hz, 1H), 3.56 (dd, *J* = 11.5, 3.6 Hz, 1H), 2.95 – 2.87 (m, 1H), 2.75 (d, *J* = 13.7 Hz, 1H), 2.55 (td, *J* = 15.4, 13.2, 5.2 Hz, 1H), 2.49 – 2.43 (m, 1H), 2.31 (d, *J* = 13.7 Hz, 1H), 2.08 – 2.04 (m, 1H), 1.99 (d, *J* = 14.5 Hz, 1H), 1.93 (d, *J* = 5.1 Hz, 2H), 1.91 (d, *J* = 15.7 Hz, 2H), 1.76 (d, *J* = 13.8 Hz, 2H), 1.72 (dd, *J* = 13.2, 4.2 Hz, 1H), 1.62 (d, *J* = 3.9 Hz, 1H), 1.61 (d, *J* = 3.8 Hz, 1H), 1.23 (t, *J* = 7.0 Hz, 1H). **$^{13}$C NMR** (125 MHz, $CD_3OD$) $\delta_C$ 169.7, 65.1, 65.1, 61.4, 57.0, 44.2, 44.2, 35.1, 33.7, 33.7, 27.3, 25.5, 25.5, 25.5, 24.7.

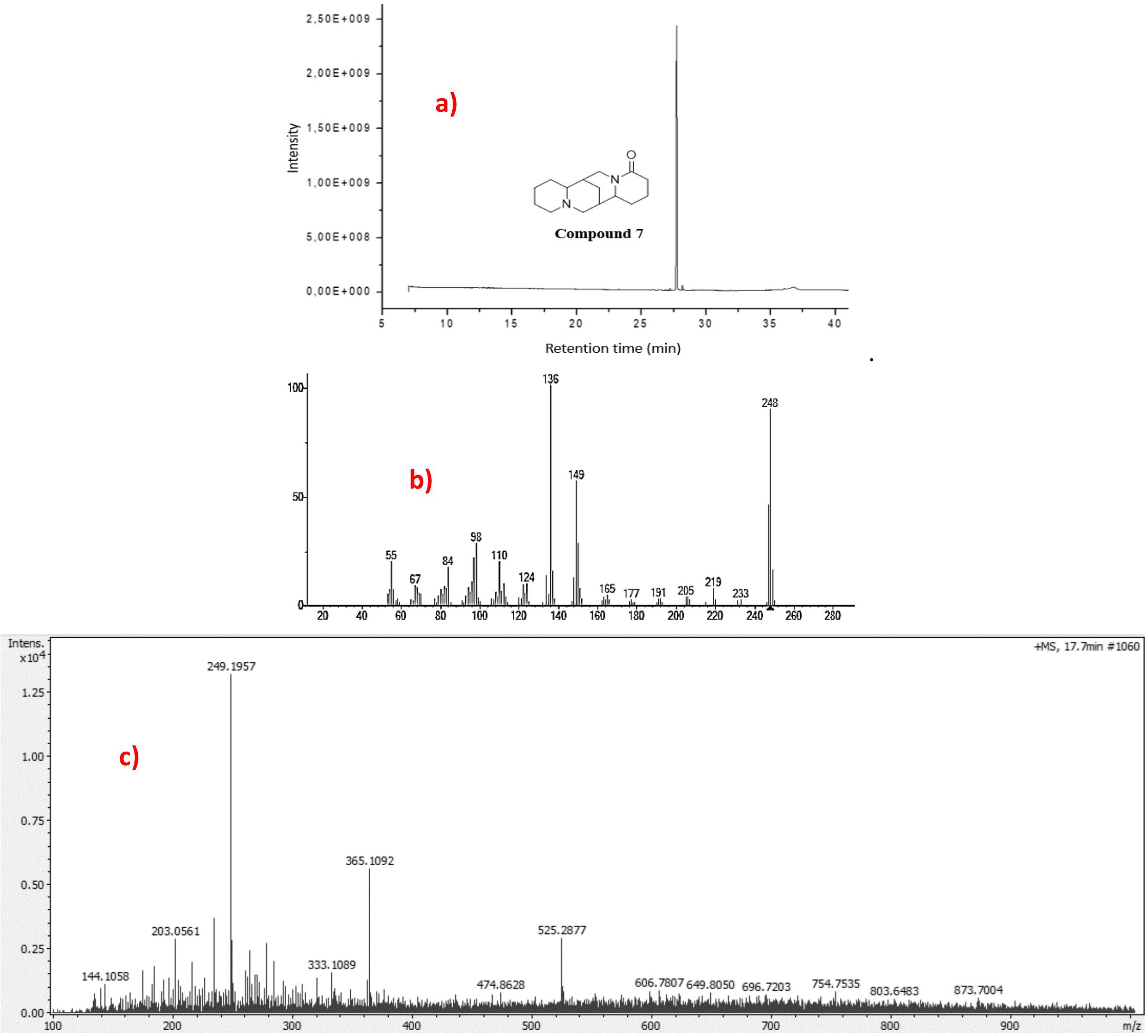


**Figure S8. a)**. Chromatographic profile obtained by GC-MS of **7**; **b)**. Mass spectra by electron impact at 70eV of **7**; **c)**. Mass spectrum by HRESIMS of **7**.

## 2. Compounds 8-10: Sparteine-type

Compound **8-10** were isolated from the leaves of *L. mutabilis* and *G. momspessulana*. Compounds **8-10** are related to those of the lupanine type because they are bridged tetracycles; however, they differ structurally due to the absence of the carbonyl group in compound **8**. In the case of compounds **9** and **10** they contain carbonyl group in positions 4 (ring A) and 10 (ring B) respectively.

### 2.1. Compound 8: (–)-sparteine

Compound **8** ($[\alpha]_D^{20}$ = –20, MeOH, *c* 0.01) was a brown oil (103.7 mg), positive for Dragendorff's reagent, and soluble in chloroform, methanol, and water. The analysis by GC-MS afforded an *m/z* = 234 that corresponds to the molecular formula $C_{15}H_{26}N_2$. This information was confirmed by HRESIMS, and a $[M+H]^+$ = 235.2184 (calcd 235.2174) was obtained. The structure of compound **8** was confirmed by $^1$H and $^{13}$C NMR, whose signals were compared with the literature and agreed with the data reported for (–)-sparteine, isolated from *L. albus* [1,9].

**$^1$H NMR** (500 MHz, $CDCl_3$) $\delta_H$ 2.64 (d, J = 6.0 Hz, 1H), 2.62 (dd, J = 17.9, 3.9 Hz, 1H), 2.57 (dd, J = 8.6, 2.0 Hz, 1H), 2.49 – 2.43 (m, 1H), 2.19 (dd, J = 11.1, 2.3 Hz, 1H), 2.05 – 2.01 (m, 1H), 1.96 (dd, J = 11.4, 3.9 Hz, 1H), 1.94 (dd, J = 10.8, 4.5 Hz, 1H), 1.92 – 1.89 (m, 1H), 1.88 – 1.86 (m, 1H), 1.85 (d, J = 2.9 Hz, 1H), 1.76 (d, J = 1.8 Hz, 1H), 1.70 – 1.67 (m, 2H), 1.53 – 1.50 (m, 2H), 1.48 – 1.45 (m, 1H), 1.45 – 1.41 (m, 1H), 1.34 (dd, J = 11.5, 4.2 Hz, 1H), 1.36 – 1.29 (m, 2H), 1.28 – 1.22 (m, 2H), 1.22 – 1.15 (m, 1H), 1.15 – 1.10 (m, 1H). **$^{13}$C NMR** (125 MHz, $CDCl_3$) $\delta_C$ 70.6, 66.2, 62.3, 59.8, 57.2, 56.2, 53.8, 34.5, 33.9, 29.4, 29.2, 27.9, 24.5, 24.3, 23.1.

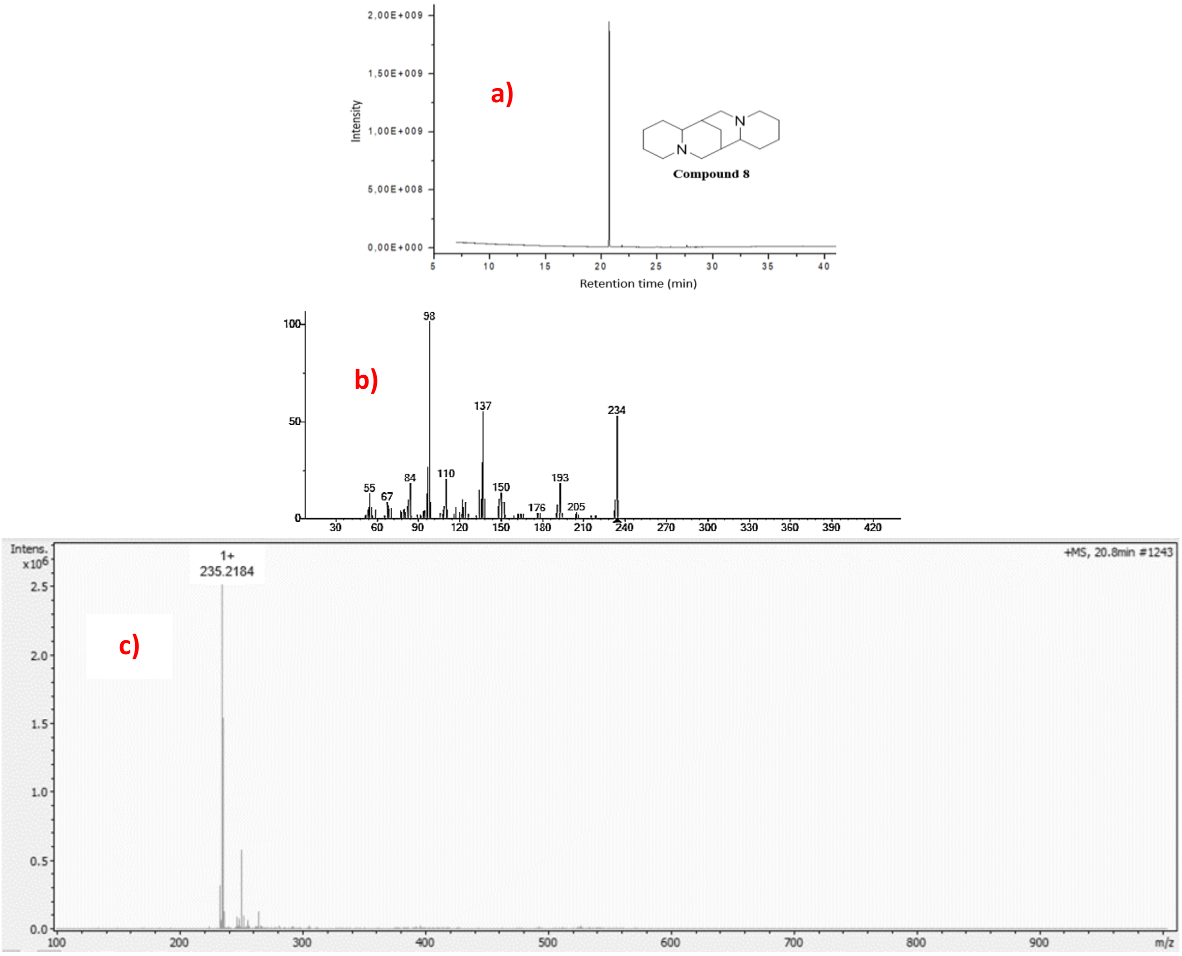


**Figure S9. a)**. Chromatographic profile obtained by GC-MS of **8**; **b)**. Mass spectra by electron impact at 70eV of **8**; **c)**. Mass spectrum by HRESIMS of **8**.

## 2.2. Compound 9: (+)-aphylline

Compound **9** ($[\alpha]_D^{20}$ = +15, MeOH, *c* 0.01) was a brown oil (1407.4 mg), positive for Dragendorff's reagent, soluble in chloroform, methanol, and water. The analysis by GC-MS afforded an *m/z* = 248 that corresponds to the molecular formula $C_{15}H_{24}N_2O$. This information was confirmed by HRESIMS, and a $[M+H]^+$ 249.1956 (calcd 249.1966) was obtained. The structure of compound **9** was confirmed by $^1H$ and $^{13}C$ NMR, whose signals were compared with the literature and agreed with the data reported for (+)-aphylline [7,8].

**$^1$H NMR** (500 MHz, $CD_3OD$) $\delta_H$ 4.69 (ddt, J = 13.2, 4.3, 2.2 Hz, 1H), 3.42 – 3.37 (m, 1H), 3.18 (dd, J = 11.9, 3.0 Hz, 1H), 3.05 (dt, J = 12.4, 2.6 Hz, 1H), 2.83 – 2.79 (m, 2H), 2.66 – 2.62 (m, 1H), 2.50 (td, J = 13.0, 3.1 Hz, 1H), 2.32 (t, J = 2.9 Hz, 1H), 2.09 – 2.05 (m, 1H), 2.04 – 2.02 (m, 1H), 1.99 (dd, J = 12.9, 4.0 Hz, 1H), 1.96 – 1.89 (m, 2H), 1.78 – 1.71 (m, 2H), 1.70 (tt, J = 3.3, 1.8 Hz, 1H), 1.67 (dddd, J = 10.7, 7.4, 3.8, 1.6 Hz, 2H), 1.62 – 1.51 (m, 2H), 1.44 (tdd, J = 12.9, 4.3, 3.5 Hz, 1H), 1.21 – 1.15 (m, 2H). **$^{13}$C NMR** (125 MHz, $CD_3OD$) $\delta_C$ 174.7, 60.8, 55.3, 49.0, 47.5, 45.4, 43.9, 33.9, 30.1, 26.8, 26.1, 25.7, 23.7, 23.5, 19.8.

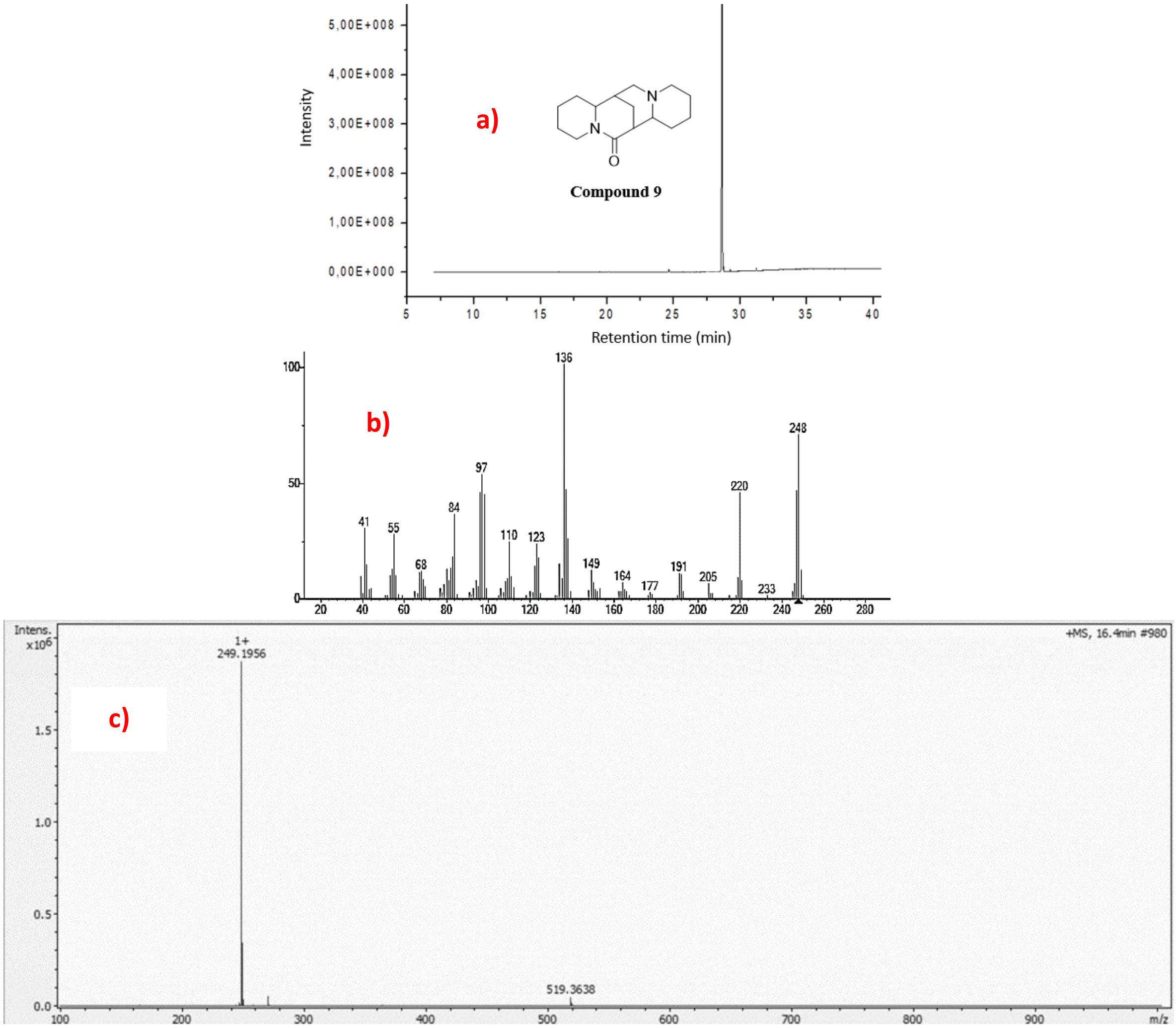


**Figure S10. a**). Chromatographic profile obtained by GC-MS of **9**; **b**). Mass spectra by electron impact at 70eV of **9**; **c**). Mass spectrum by HRESIMS of **9**.

## 2.3. Compound 10: (–)-multiflorine

Compound **10** ($[\alpha]_D^{20}$ = –42, MeOH, *c* 0.01) was a yellow oil (29.3 mg), positive for Dragendorff's reagent, soluble in chloroform, methanol, and water. The analysis by GC-MS afforded an *m/z* = 246 that corresponds to the molecular formula $C_{15}H_{22}N_2O$. This information was confirmed by HRESIMS, and a $[M + H]^+$ = 247.1801 (calcd 247.1810) was obtained. The structure of compound **10** was confirmed by $^1$H and $^{13}$C NMR, whose signals were compared with the literature and agreed with the data reported for (–)-multiflorine [10,11].

**$^1$H NMR** (400 MHz, $CD_3OD$) $\delta_H$ 7.09 (d, *J* = 8.0 Hz, 1H), 5.86 (dd, *J* = 36.8, 5.5 Hz, 1H), 3.63 (d, *J* = 10.3 Hz, 1H), 3.23 (d, *J* = 19.9 Hz, 1H), 3.08 (d, *J* = 12.8 Hz, 1H), 2.85 (d, *J* = 2.2 Hz, 2H), 2.82 – 2.79 (m, 1H), 2.75 (d, *J* = 12.2 Hz, 1H), 2.16 (d, *J* = 14.2 Hz, 4H), 2.04 (dd, *J* = 13.8, 11.2 Hz, 2H), 1.83 (d, *J* = 4.0 Hz, 1H), 1.76 (dd, *J* = 6.8, 5.6 Hz, 1H), 1.63 (d, *J* = 4.6 Hz, 1H), 1.61 (d, *J* = 4.5 Hz, 1H), 1.58 – 1.48 (m, 1H), 1.29 (d, *J* = 9.3 Hz, 2H). **$^{13}$C NMR** (125 MHz, $CD_3OD$) $\delta_C$ 192.3, 155.4, 98.8, 63.5, 60.3, 57.5, 55.2, 51.1, 39.4, 34.6, 31.4, 31.1, 25.8, 24.9, 23.8.

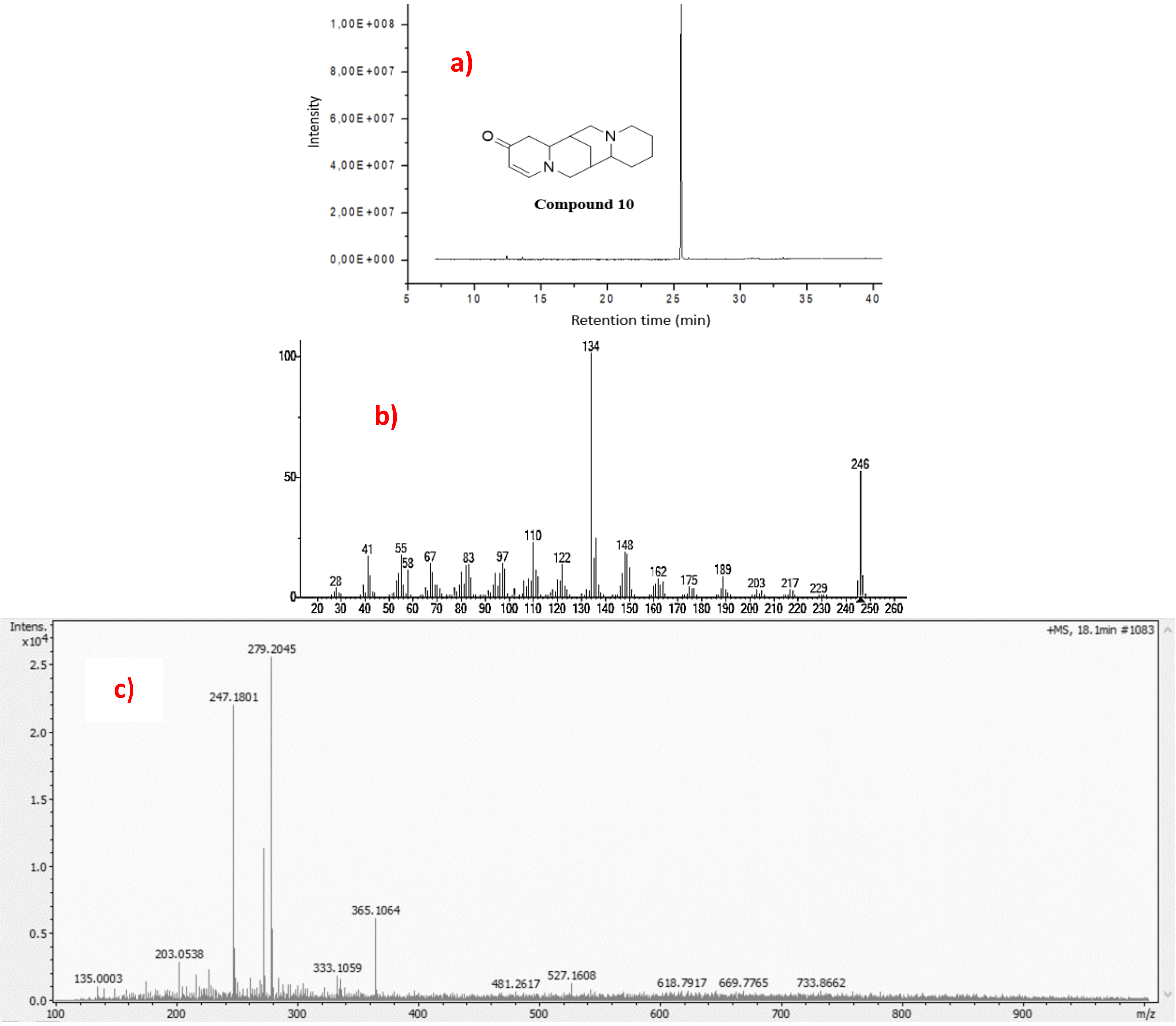


**Figure S11**. **a**). Chromatographic profile obtained by GC-MS of **10**; **b**). Mass spectra by electron impact at 70eV of **10**; **c**). Mass spectrum by HRESIMS of **10**.

## 3. <u>Compound 11: Lupinine-type</u>

Compound **11** was isolated from the leaves of *L. polyphyllus polyphyllus 'rusell'*. Compound **11**, a lupinine-type QA, is characterized by an azabicyclic moiety, whose main building block is a quinolizidine.

### 3.1. Compound 11: (–)-lupinine

Compound **11** ($[\alpha]_D^{20}$ = –63, MeOH, *c* 0.02) was a white solid (10.1 mg, mp. 68-70 °C), positive for Dragendorff's reagent, soluble in chloroform, methanol, and water. The analysis by GC-MS afforded an *m/z* = 169 that corresponds to the molecular formula $C_{10}H_{19}NO$. This information was confirmed by HRESIMS, and a $[M+H]^+$ = 170.1547 (calcd 170.1544) was obtained. The structure of compound **11** was confirmed by $^1$H and $^{13}$C NMR, whose signals were compared with the literature and agreed with the data reported for (–)-lupinine, isolated from *L. luteus* [12,13]**.**

**$^1$H NMR** (500 MHz, $CDCl_3$) $\delta_H$ 4.15 (ddd, J = 10.7, 4.7, 1.4 Hz, 1H), 3.69 (d, J = 10.6 Hz, 1H), 2.84 – 2.78 (m, 2H), 2.20 – 2.13 (m, 1H), 2.11 (d, J = 11.0 Hz, 1H), 2.03 – 1.97 (m, 1H), 1.83 (td, J = 11.9, 3.1 Hz, 2H), 1.79 – 1.75 (m, 1H), 1.74 – 1.70 (m, 1H), 1.59 (dd, J = 4.9, 1.6 Hz, 1H), 1.56 (dd, J = 6.4, 2.0 Hz, 1H), 1.55 – 1.52 (m, 2H), 1.50 (dd, J = 12.7, 3.5 Hz, 2H), 1.29 – 1.20 (m, 1H). **$^{13}$C NMR** (125 MHz, , $CDCl_3$) $\delta_C$ 66.2, 65.3, 57.3, 57.2, 38.3, 31.6, 29.9, 25.8, 24.8, 23.1.

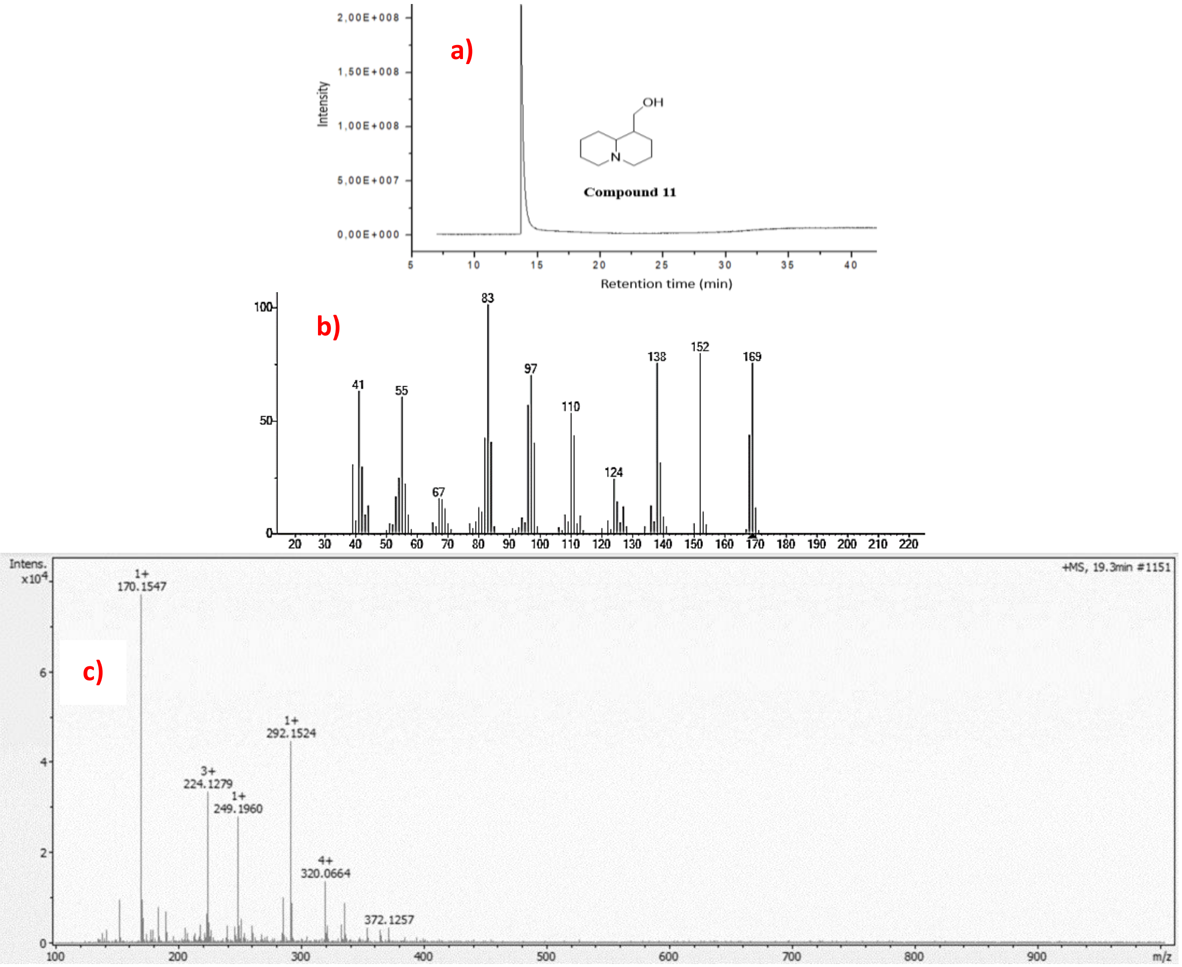


**Figure S12. a**). Chromatographic profile obtained by GC-MS of **11**; **b**). Mass spectra by electron impact at 70eV of **11**; **c**). Mass spectrum by HRESIMS of **11**.

## 4. Compounds 12-15: Cytisine-type

Compounds **12-15** were isolated from the leaves of *G. monspessulana*. Each cytisine-type QA was structurally characterized by the presence of a bridged tricycle with a 2-pyridone in the A ring. Compounds **13** and **14** were structurally related to compound **12**, but these differ by the presence of a substitution in the nitrogen at position 12 (methyl and formyl). In case of compound **15** it is related to compound 1 by being a bridged tetracycle and it is also related to compound 12 by the presence of the 2-pyridone in the A-ring.

### 4.1. Compounds 12: (–)-cytisine

The compound **12** ($[\alpha]^{20}_{D}$ = –128, MeOH, *c* 0.02) was a white solid (264.2 mg, mp. 153-154 °C), positive for Dragendorff's reagent, soluble in methanol, ethanol, and water. The analysis by GC-MS afforded an *m/z* = 190 that corresponds to the molecular formula $C_{11}H_{14}N_2O$. This information was confirmed by HRESIMS, and a $[M+H]^+$ = 191.1176 (calcd 191.1184) was obtained. The structure of compound **12** was confirmed by $^1$H and $^{13}$C NMR, whose signals were compared with the literature and agreed with the data reported for (–)-cytisine [14].

**$^1$H NMR** (500 MHz, $CD_3OD$) $\delta_H$ 7.54 (dd, J = 9.1, 6.9 Hz, 1H), 6.52 (d, J = 9.1 Hz, 1H), 6.44 (dd, J = 6.9, 0.6 Hz, 1H), 4.21 (d, J = 15.8 Hz, 1H), 4.02 (dd, J = 15.9, 6.8 Hz, 1H), 3.52 – 3.42 (m, 4H), 3.41 (s, 1H), 2.80 (s, 1H), 2.10 (m, 2H). **$^{13}$C NMR** (125 MHz, $CD_3OD$) $\delta_C$ 165.6, 148.4, 141.6, 118.7, 109.1, 51.0, 49.8, 49.0, 33.3, 26.7, 24.3.

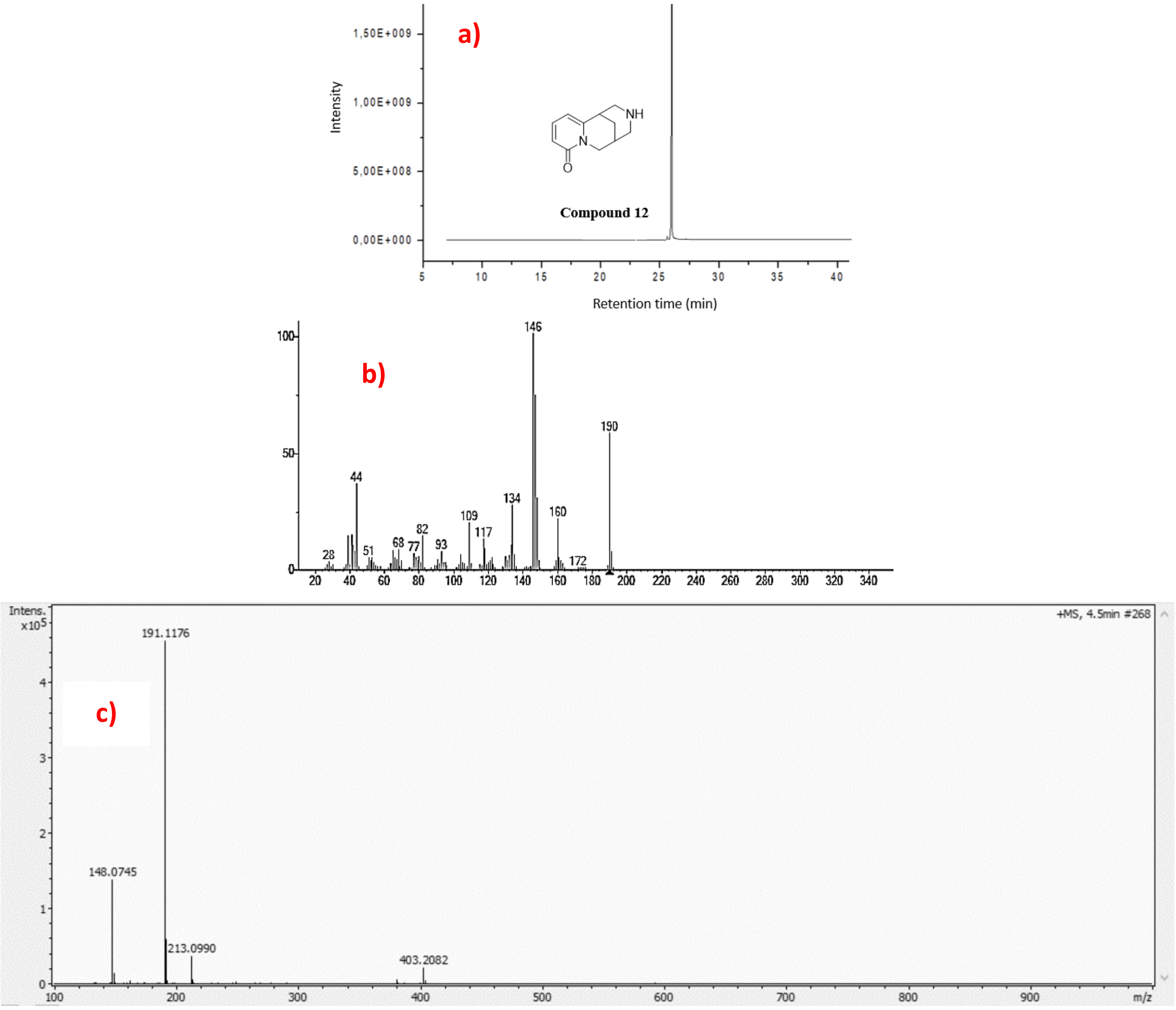


**Figure S13.** a). Chromatographic profile obtained by GC-MS of **12**; b). Mass spectra by electron impact at 70eV of **12**; c). Mass spectrum by HRESIMS of **12**.

## 4.2. Compound 13: (–)-*N*-methylcytisine

Compound **13** ($[\alpha]_D^{20}$ = –256, MeOH, *c* 0.03) was a white crystalline solid (23 mg, mp. 138-139 °C), positive for Dragendorff's reagent, soluble in methanol, ethanol, and water. The analysis by GC-MS afforded an *m/z* = 204 that corresponds to the molecular formula $C_{12}H_{16}N_2O$. This information was confirmed by HRESIMS, and a $[M+H]^+$ = 205.1326 (calcd 205.1340) was obtained. The structure of compound **13** was confirmed by $^1$H and $^{13}$C NMR, whose signals were compared with the literature and agreed with the data reported for (–)-*N*-methylcytisine [14].

**$^1$H NMR** (500 MHz, $CDCl_3$) $\delta_H$ 7.27 (dd, *J* = 8.9, 7.0 Hz, 1H), 6.43 (dd, *J* = 9.0, 1.4 Hz, 1H), 5.98 (dd, *J* = 6.9, 1.1 Hz, 1H), 4.04 (d, *J* = 15.4 Hz, 1H), 3.89 (dd, *J* = 15.0, 7.3 Hz, 1H), 2.95 – 2.91 (m, 1H), 2.88 (ddd, *J* = 11.0, 2.9, 1.5 Hz, 1H), 2.82 (ddt, *J* = 10.7, 3.3, 1.6 Hz, 1H), 2.44 – 2.39 (m, 1H), 2.23 (ddd, *J* = 12.0, 10.9, 1.6 Hz, 2H), 2.12 (s, 3H), 1.88 – 1.70 (m, 2H). **$^{13}$C NMR** (125 MHz, $CDCl_3$) $\delta_C$ 165.7, 153.5, 141.2, 116.7, 107.8, 63.6, 63.1, 51.5, 49.0, 46.5, 36.6, 29.3, 25.9.

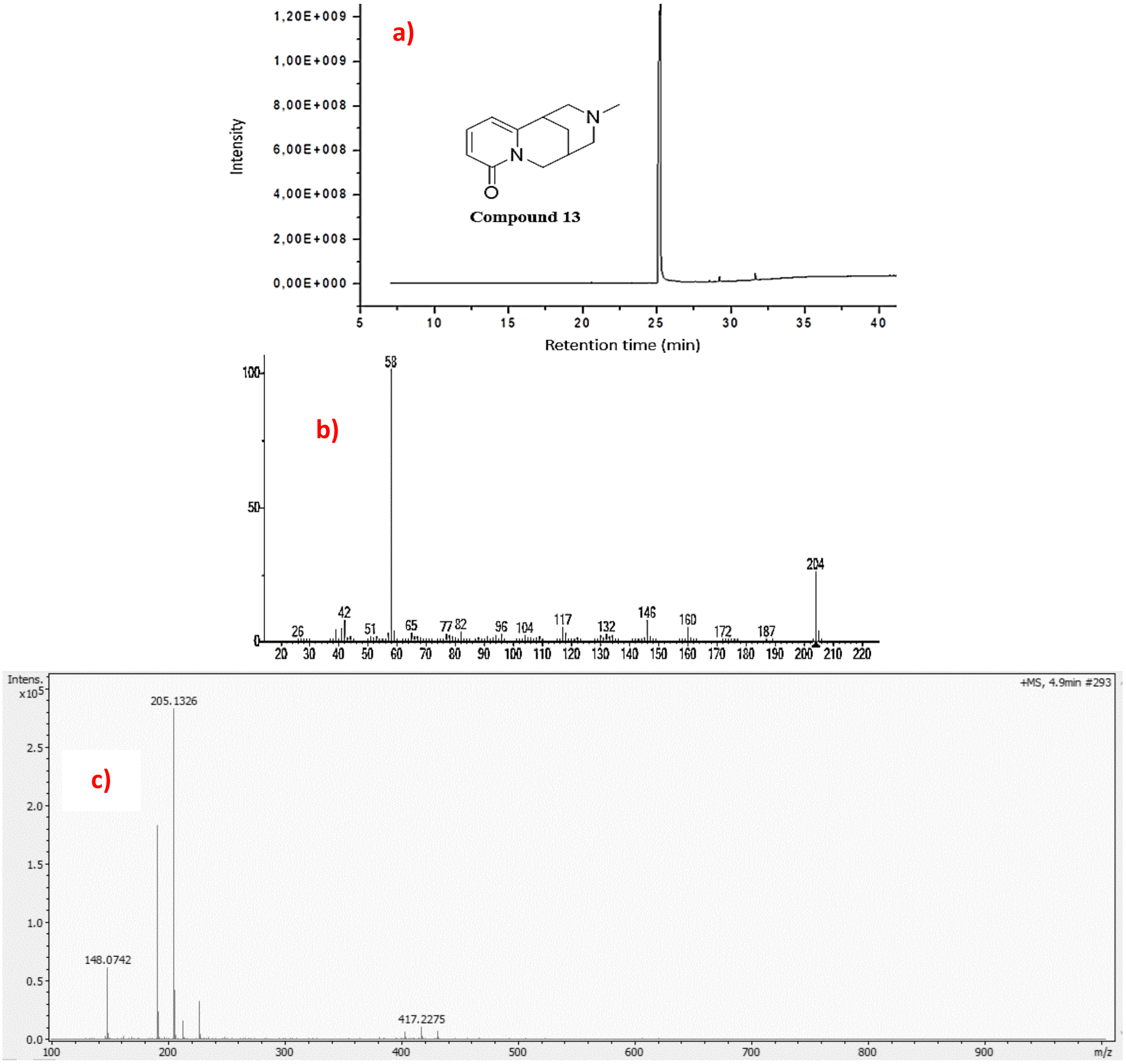


**Figure S14. a**). Chromatographic profile obtained by GC-MS of **13**; **b**). Mass spectra by electron impact at 70eV of **13**; **c**). Mass spectrum by HRESIMS of **13**.

## 4.3. Compound 14: (–)-*N*-formylcytisine

Compound **14** ($[\alpha]_D^{20}$ = –86, MeOH, *c* 0.02) was a white solid (14.2 mg, mp. 166-168 °C), positive for Dragendorff's reagent, soluble in chloroform and methanol. The analysis by GC-MS afforded an *m/z* = 218 that corresponds to the molecular formula $C_{12}H_{14}N_2O_2$. This information was confirmed by HRESIMS, and a $[M+H]^+$ = 219.1121 (calcd 219.1133) was obtained. The structure of compound **14** was confirmed by $^1$H and $^{13}$C NMR, whose signals were compared with the literature and agreed with the data reported for (–)-*N*-formylcytisine [14,15]**.**

**$^1$H NMR** (500 MHz, $CD_3OD$) $\delta_H$ 7.61 (s, 1H), 7.47 (ddd, J = 21.4, 9.0, 7.0 Hz, 1H), 6.42 (ddd, J = 9.0, 4.1, 1.3 Hz, 1H), 6.36 – 6.34 (m, 1H), 4.12 – 4.08 (m, 1H), 3.89 (ddd, J = 15.8, 6.6, 1.3 Hz, 1H), 3.86 (dd, J = 6.7, 1.2 Hz, 1H), 3.57 (dd, J = 13.0, 2.3 Hz, 1H), 3.51 – 3.47 (m, 1H), 3.07 (dd, J = 12.9, 2.6 Hz, 1H), 3.04 – 3.00 (m, 1H), 2.56 (s, 1H), 2.20 – 2.10 (m, 2H). **$^{13}$C NMR** (125 MHz, $CD_3OD$) $\delta_C$ 190.3, 179.4, 160.7, 160.2, 149.2, 134.8, 119.0, 117.8, 115.4, 77.2, 61.8.

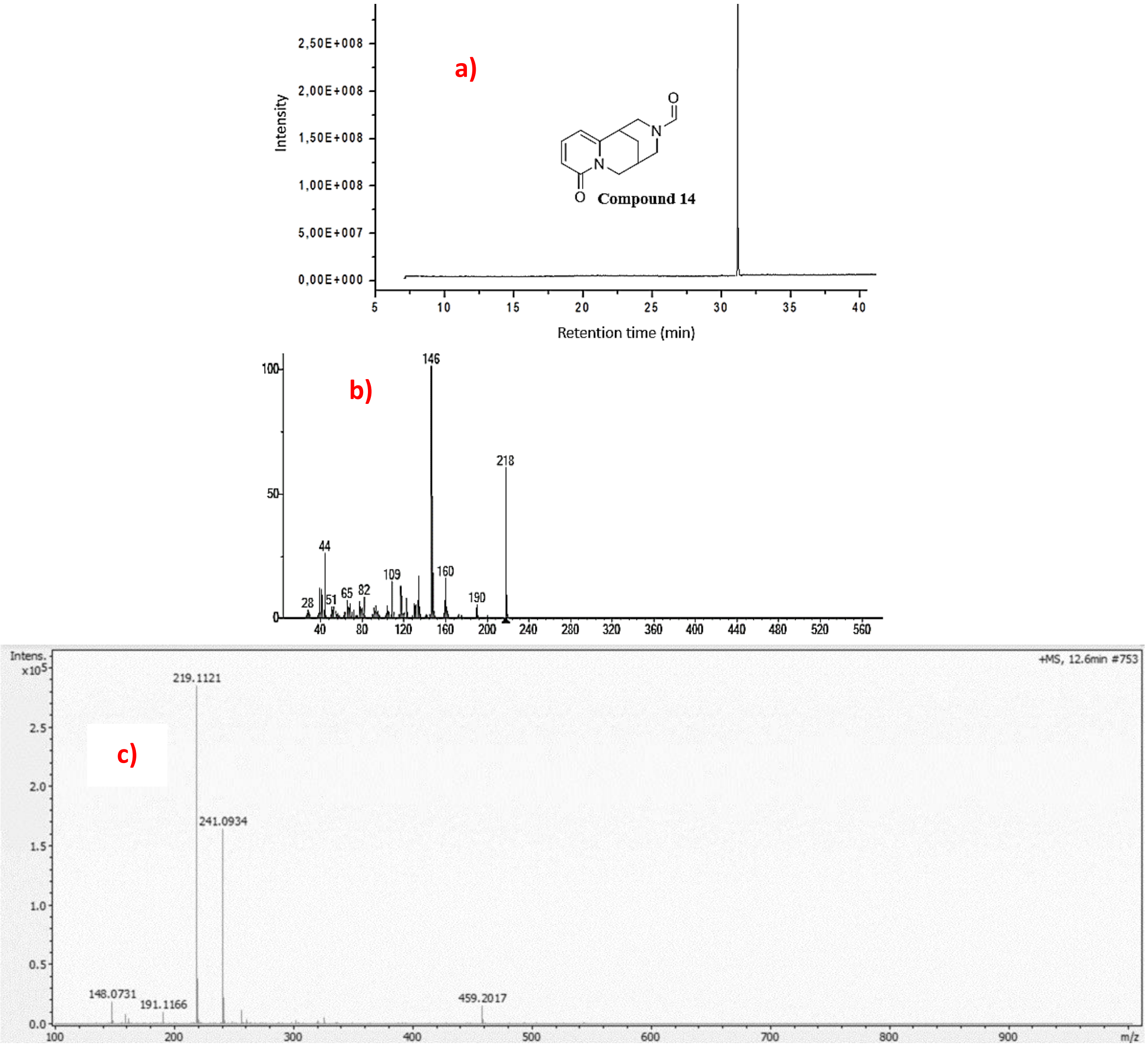


**Figure S15. a**). Chromatographic profile obtained by GC-MS of **14**; **b**). Mass spectra by electron impact at 70eV of **14**;**c**). Mass spectrum by HRESIMS of **14**.

## 4.4. Compound 15: (–)-anagyrine

Compound **15** ($[\alpha]_D^{20}$ = –172, MeOH, *c* 0.02) was a brown solid (32.1 mg, mp. 282-283 °C), positive for Dragendorff's reagent, soluble in chloroform and methanol. The analysis by GC-MS afforded an *m/z* = 244 that corresponds to the molecular formula $C_{15}H_{20}N_2O$. This information was confirmed by HRESIMS, and a $[M+H]^+$ = 245.1650 (calcd 245.1653) was obtained. The structure of compound **15** was confirmed by $^1$H and $^{13}$C NMR, whose signals were compared with the literature and agreed with the data reported for (–)-anagryrine [16].

**$^1$H NMR** (500 MHz, $CDCl_3$) $\delta_H$ 7.27 (dd, *J* = 9.2, 6.7 Hz, 1H), 6.43 (dd, *J* = 9.0, 1.3 Hz, 1H), 5.97 (dd, *J* = 6.9, 1.1 Hz, 1H), 4.06 (d, *J* = 15.4 Hz, 1H), 3.89 (dd, *J* = 15.4, 6.7 Hz, 1H), 3.72 (q, *J* = 7.0 Hz, 1H), 3.40 – 3.35 (m, 1H), 2.96 (d, *J* = 2.1 Hz, 1H), 2.88 (d, *J* = 12.0 Hz, 1H), 2.72 (td, *J* = 13.3, 2.9 Hz, 1H), 2.46 (ddd, *J* = 10.7, 2.6, 1.6 Hz, 1H), 2.16 (dd, *J* = 7.0, 4.4 Hz, 1H), 2.01 (d, *J* = 13.0 Hz, 1H), 1.88 (td, *J* = 16.1, 4.0 Hz, 2H), 1.70 – 1.65 (m, 1H), 1.64 – 1.56 (m, 1H), 1.54 – 1.44 (m, 1H), 1.24 (t, *J* = 7.0 Hz, 1H), 1.16 (d, *J* = 13.8 Hz, 1H). **$^{13}$C NMR** (125 MHz, $CDCl_3$) $\delta_C$ 165.7, 152.5, 141.3, 117.3, 108.0, 64.4, 55.3, 53.5, 52.3, 36.2, 33.7, 25.6, 23.8, 20.8, 19.8.

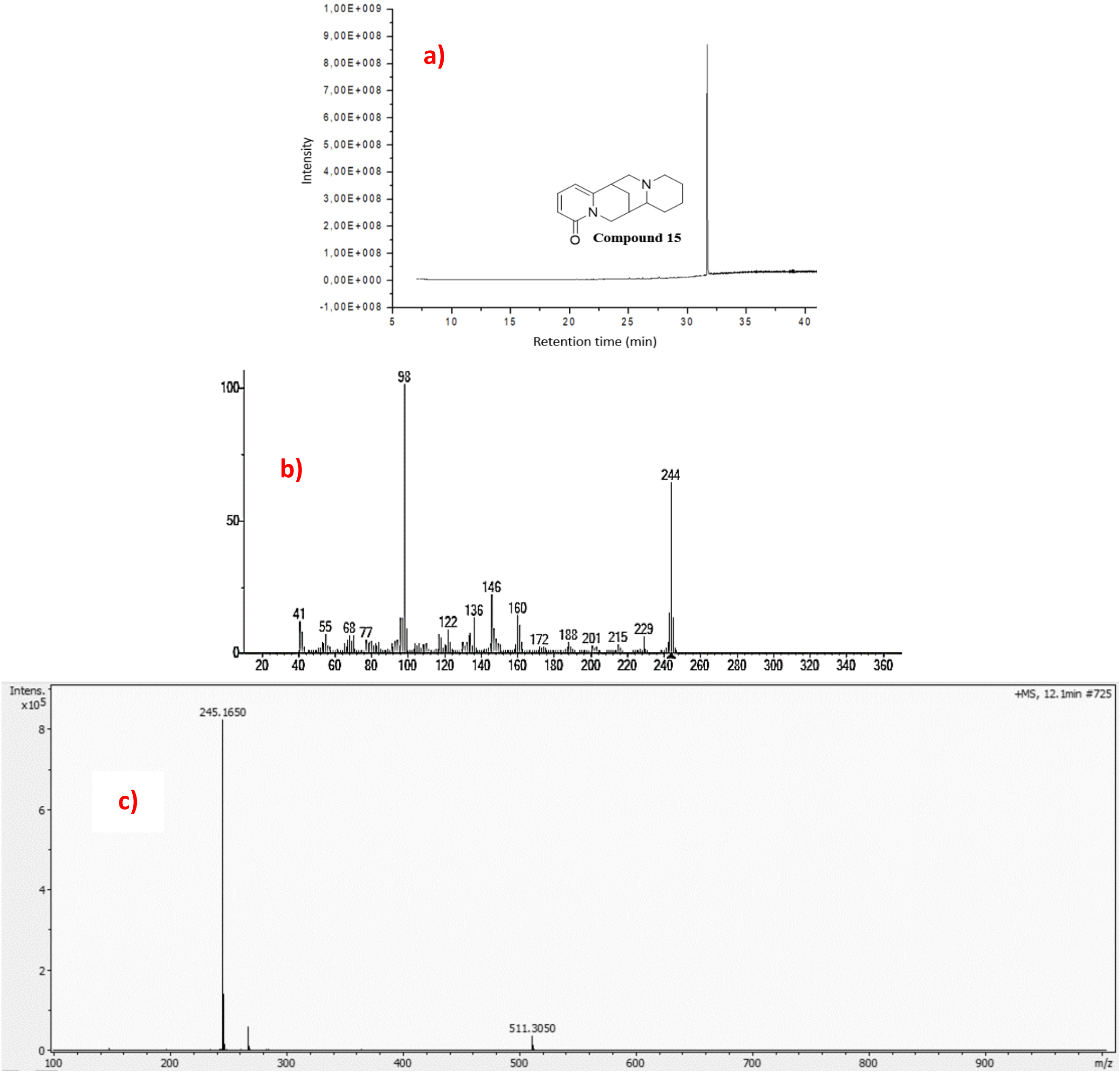


**Figure S16. a**). Chromatographic profile obtained by GC-MS of **15**; **b**). Mass spectra by electron impact at 70eV of **15**; **c**). Mass spectrum by HRESIMS of **15**.

## 5. Compounds 16-17: Tetrahydrocytisine type

Compounds **16-17** were isolated from the leaves of *L. polyphyllus 'rusell'*. Each tetrahydrocytisine-type QA was structurally characterized by the presence of a bridged tricycle with a lactam group in the A ring. Compounds **16** and **17** were structurally related to compound **12**, for being tricyclic, but these differ by the absence of the 2-pyridone from ring A.

### 5.1. Compound 16: (–)-tetrahydrorhombifoline

Compound **16** ($[\alpha]_D^{20}$ = –210, MeOH, *c* 0.02) was a yellow oil (55.8 mg), positive for Dragendorff's reagent, soluble in dichloromethane, chloroform, and methanol. The analysis by GC-MS afforded an *m/z* = 248 that corresponds to the molecular formula $C_{15}H_{24}N_2O$. This information was confirmed by HRESIMS, and a $[M+H]^+$ = 249.1966 (calcd 249.1966) was obtained. The structure of compound **16** was confirmed by $^1H$ and $^{13}C$ NMR, whose signals were compared with the literature and agreed with the data reported for (–)-tetrahydrorombifoline [17].

**$^1$H NMR** (500 MHz, $CDCl_3$) $\delta_H$ 5.78 – 5.66 (m, 1H), 4.97 (dd, J = 17.4, 0.8 Hz, 1H), 4.91 (d, J = 10.4 Hz, 1H), 4.62 (d, J = 13.6 Hz, 1H), 3.42 (dd, J = 9.0, 4.3 Hz, 1H), 3.11 (d, J = 11.4 Hz, 1H), 2.91 (d, J = 10.7 Hz, 1H), 2.76 (d, J = 13.6 Hz, 1H), 2.39 (d, J = 2.4 Hz, 1H), 2.36 (d, J = 2.4 Hz, 1H), 2.28 – 2.22 (m, 2H), 2.21 – 2.16 (m, 2H), 2.14 – 2.09 (m, 2H), 2.01 (d, J = 10.8 Hz, 1H), 1.84 – 1.79 (m, 2H), 1.75 – 1.70 (m, 2H), 1.69 – 1.65 (m, 1H), 1.65 – 1.58 (m, 2H). **$^{13}$C NMR** (125 MHz, $CDCl_3$) $\delta_C$ 168.9, 137.0, 115.1, 59.2, 58.8, 58.2, 54.0, 46.3, 34.0, 33.4, 33.0, 31.4, 29.2, 27.9, 20.1.

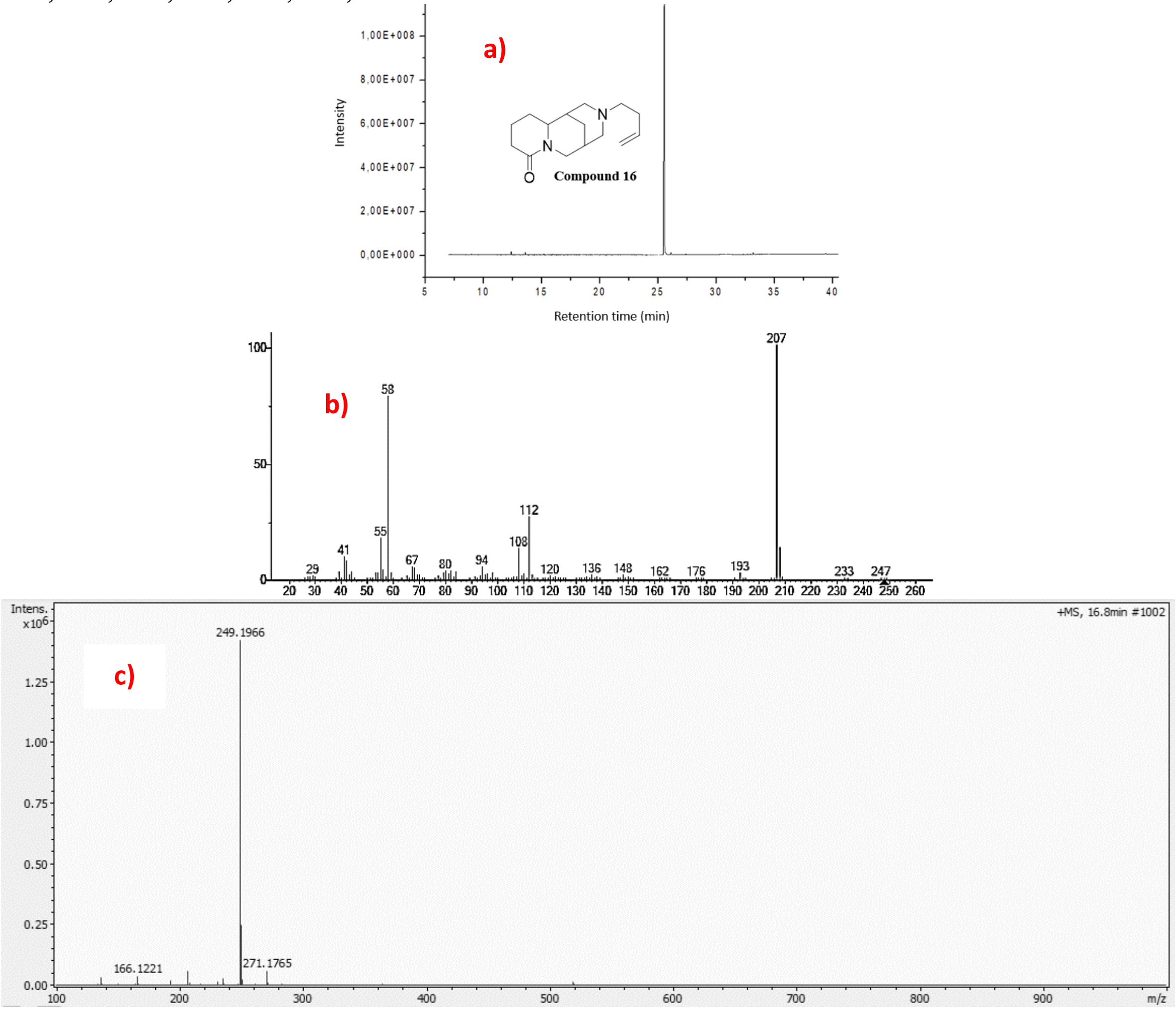


**Figure S17. a**). Chromatographic profile obtained by GCMS of **16**; **b**). Mass spectra by electron impact at 70eV of **16**; **c**). Mass spectrum by HRESIMS of **16.**

## 5.2. Compound 17: (–)-angustifoline

Compound **17** ($[\alpha]_D^{20}$ = –7.5, MeOH, *c* 0.01) was a brown solid (32.5 mg, mp. 80-82 °C), positive for Dragendorff's reagent, soluble in chloroform ,methanol and water. The analysis by GC-MS afforded an *m/z* = 234 that corresponds to the molecular formula $C_{14}H_{22}N_2O$. This information was confirmed by HRESIMS, and a $[M+H]^+$ = 235.1804 (calcd 235.1810) was obtained. The structure of compound **17** was confirmed by $^1$H and $^{13}$C NMR, whose signals were compared with the literature and agreed with the data reported for (–)-angustifoline [17,18].

**$^1$H NMR** (500 MHz, $CDCl_3$) $\delta_H$ 5.75 (ddt, J = 24.2, 10.2, 7.1 Hz, 1H), 5.07 (dd, J = 3.3, 1.4 Hz, 1H), 5.05 – 5.02 (m, 1H), 4.62 (dt, J = 13.6, 2.2 Hz, 1H), 3.46 (ddd, J = 10.0, 5.5, 2.4 Hz, 1H), 3.03 – 2.95 (m, 2H), 2.89 (d, J = 6.7 Hz, 1H), 2.86 (dd, J = 14.0, 3.0 Hz, 1H), 2.53 – 2.47 (m, 1H), 2.46 – 2.42 (m, 1H), 2.40 – 2.36 (m, 1H), 2.31 – 2.27 (m, 1H), 2.13 – 2.09 (m, 1H), 1.91 – 1.86 (m, 1H), 1.82 (dd, J = 12.0, 2.4 Hz, 1H), 1.79 – 1.78 (m, 1H), 1.74 (dd, J = 3.8, 2.1 Hz, 1H), 1.72 – 1.68 (m, 1H), 1.63 (dd, J = 8.2, 4.0 Hz, 1H), 1.59 (d, J = 1.3 Hz, 1H), 1.52 (d, J = 3.0 Hz, 1H). **$^{13}$C NMR** (125 MHz, $CDCl_3$) $\delta_C$ 170.6, 135.9, 117.0, 60.4, 57.1, 48.0, 41.8, 37.5, 33.3, 32.7, 31.1, 28.1, 27.8, 20.2.

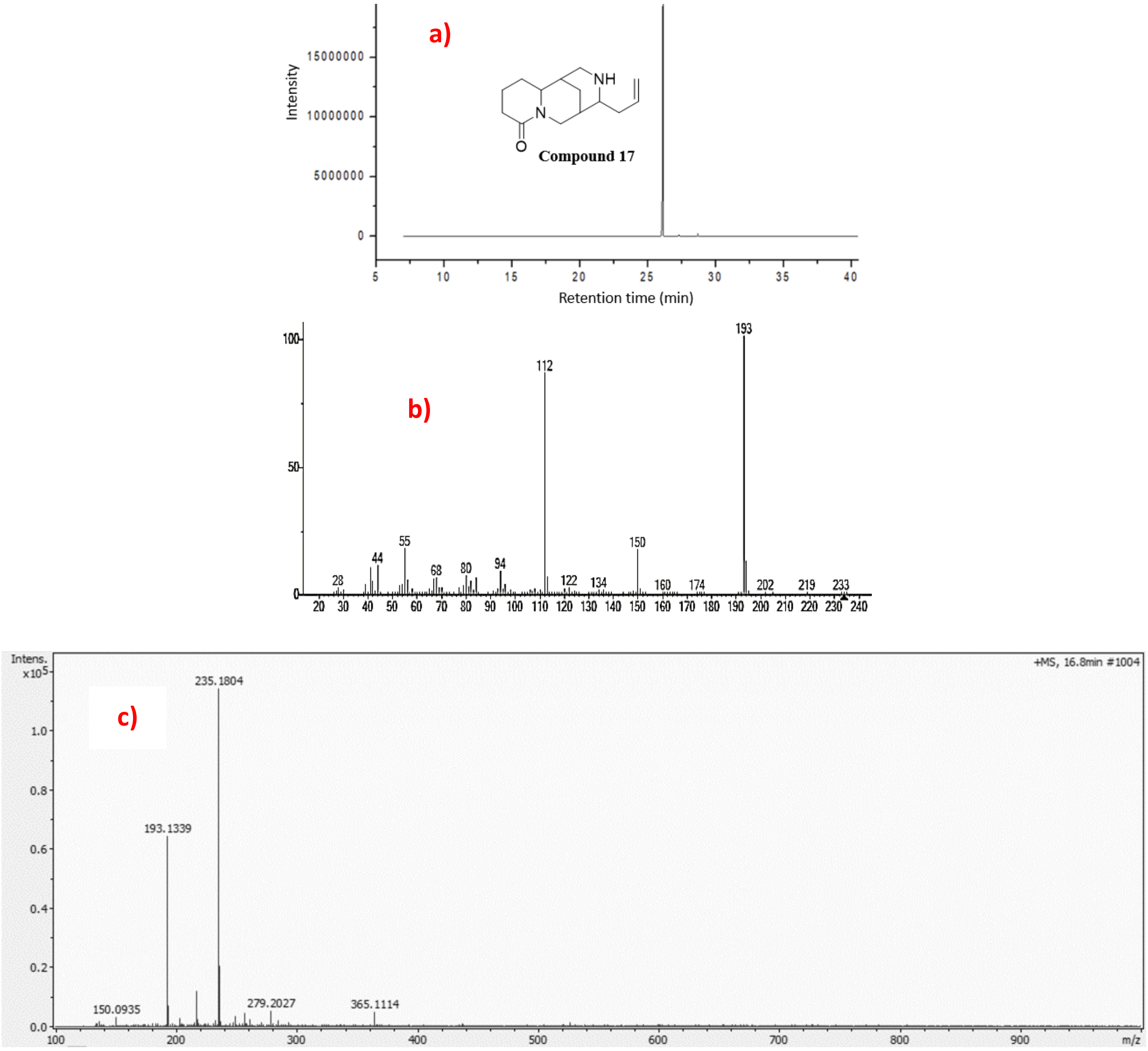


**Figure S18. a**). Chromatographic profile obtained by GC-MS of **17**; **b**). Mass spectra by electron impact at 70eV of **17**; **c**). Mass spectrum by HRESIMS of **17**.

## 6. Compounds 18-20: Matrine-type

Compounds **18-20** were isolated from the leaves of *L. polyphyllus 'rusell'* and *G. monspessulana*. Each matrine-type QA was structurally characterized by the presence of a fused tetracycle with a lactam group in the D ring. Compounds **19** and **20** were structurally related to compound **18**, but these differ by the presence of a double bond in D-ring (in **19)** and and an *N*-oxide (in **20)**.

### 6.1. Compound 18: (–)-matrine

Compound **18** ($[\alpha]_D^{20}$ = –96, MeOH, *c* 0.01) was a white solid (15.6 mg, mp. 66-68 °C), positive for Dragendorff's reagent, soluble in chloroform ,methanol, and water. The analysis by GC-MS afforded an *m/z* = 248 that corresponds to the molecular formula $C_{15}H_{24}N_2O$. This information was confirmed by HRESIMS, and a $[M+H]^+$ = 249.1972 (calcd 249.1966) was obtained. The structure of compound **18** was confirmed by $^1$H and $^{13}$C NMR, whose signals were compared with the literature and agreed with the data reported for (–)-matrine [19].

**$^1$H NMR** (500 MHz, $CDCl_3$) $\delta_H$ 4.39 – 4.33 (m, 1H), 3.83 – 3.75 (m, 1H), 3.02 (t, *J* = 12.6 Hz, 1H), 2.81 (d, *J* = 11.2 Hz, 1H), 2.75 (d, *J* = 11.4 Hz, 1H), 2.43 – 2.36 (m, 1H), 2.26 – 2.17 (m, 1H), 2.09 – 2.03 (m, 2H), 1.95 (dd, *J* = 20.5, 8.0 Hz, 2H), 1.90 – 1.83 (m, 1H), 1.79 – 1.73 (m, 1H), 1.70 (dd, *J* = 13.1, 3.4 Hz, 1H), 1.67 – 1.64 (m, *J* = 7.4 Hz, 1H), 1.64 – 1.62 (m, 1H), 1.60 – 1.57 (m, 1H), 1.57 – 1.53 (m, 1H), 1.52 – 1.46 (m, 1H), 1.41 (d, *J* = 1.8 Hz, 2H), 1.37 (dd, *J* = 9.3, 4.8 Hz, 2H). **$^{13}$C NMR** (125 MHz, $CDCl_3$) $\delta_C$ 169.6, 63.9, 57.4, 57.3, 53.3, 43.3, 41.5, 35.4, 32.9, 27.8, 27.3, 26.5, 21.2, 20.8, 19.1.

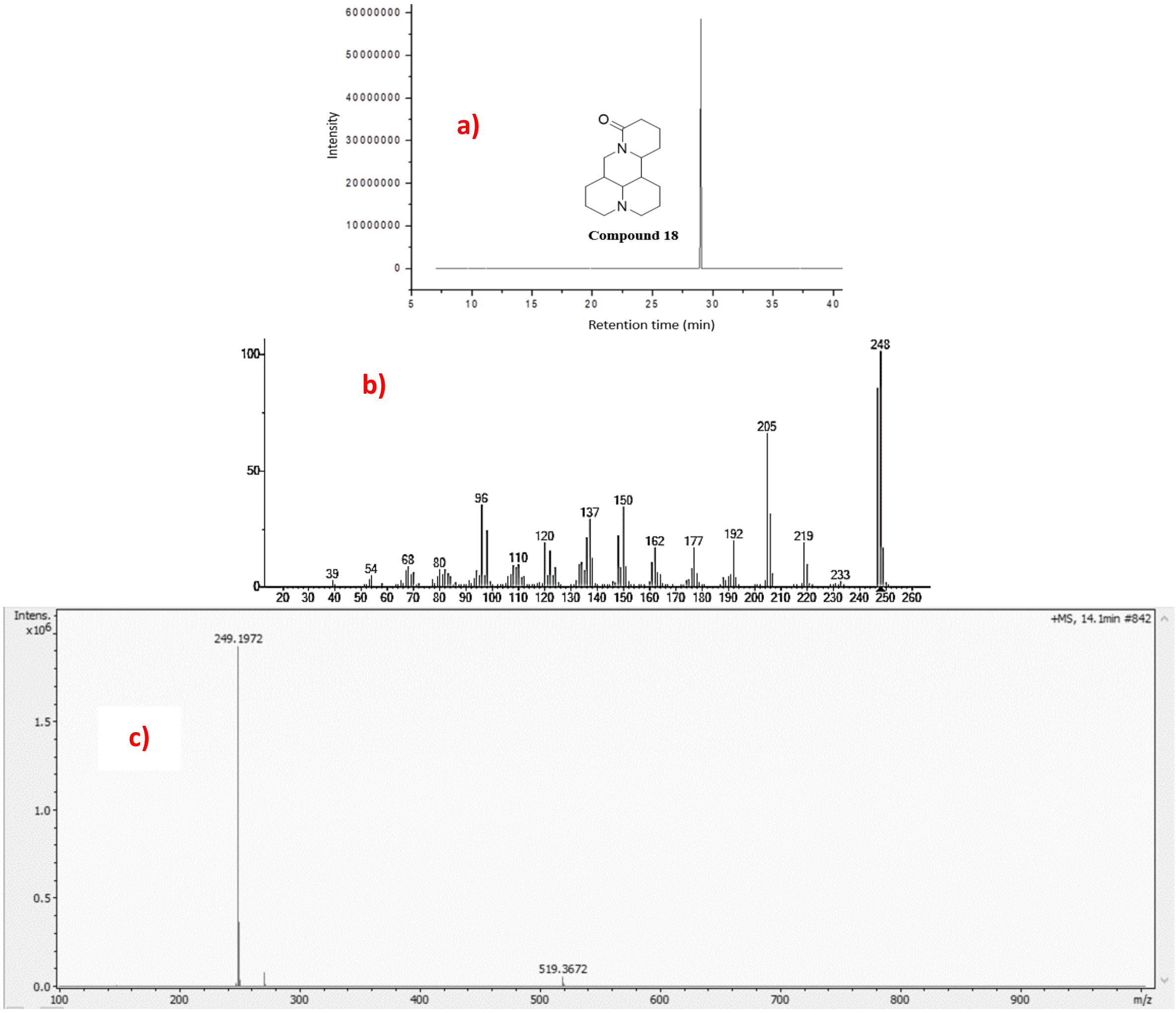


**Figure S19. a)**. Chromatographic profile obtained by GC-MS of **18**; **b)**. Mass spectra by electron impact at 70eV of **18**; **c).** Mass spectra by HRESIMS of **18**.

## 6.2. Compound 19: (+)-lehmanine

Compound **19** ($[\alpha]_D^{20}$ = +29, MeOH, *c* 0.02) was a yellow oil (16.3 mg), positive for Dragendorff's reagent, and soluble in chloroform, methanol, and water. The analysis by GC-MS afforded an *m/z* = 246 that corresponds to the molecular formula $C_{15}H_{22}N_2O$. This information was confirmed by HRESIMS, and a $[M+H]^+$ = 247.1807 (calcd 247.1810) was obtained. The structure of compound **19** was confirmed by $^1H$ and $^{13}C$ NMR, whose signals were compared with the literature and agreed with the data reported for (+)-lehmanine [20].

**$^1$H NMR** (500 MHz, $CDCl_3$) $\delta_H$ 7.06 (dt, 1H), 5.21-5.17 (m, 1H), 3.58 (ddd, *J* = 2.47, 4.60, 11.22 Hz, 1H), 3.21-3.18 (m, 1H), 2.99-2.95 (m, 1H), 2.76 (dd, *J*=1.92, 4.21 Hz, 1H), 2.71 (td, *J*= 2.93, 13.27, 13.46 Hz,1H), 2.62 (ddt, *J*=2.79, 2.79, 5.54, 7.73 Hz, 2H), 2.38-2.33 (m, 2H), 2.12-2.09 (m, 2H), 1.98 (dd, *J*= 3.98, 12.56 Hz, 2H), 1.83-1.79 (m, 2H), 1.51 (ddt, *J* = 2.99, 2.99, 5.90, 12.77 Hz, 1H), 1.64 – 1.62 (m, 1H), 1.52 – 1.50 (m, 1H), 1.17 – 1.14 (m, 2H). **$^{13}$C NMR** (125 MHz, $CDCl_3$) $\delta_C$ 173.2, 124.8, 112.3, 61.9, 59.3, 55.4, 55.4, 49.0, 47.3, 45.1, 33.6, 26.7, 26.6, 24.0, 23.5, 20.0.

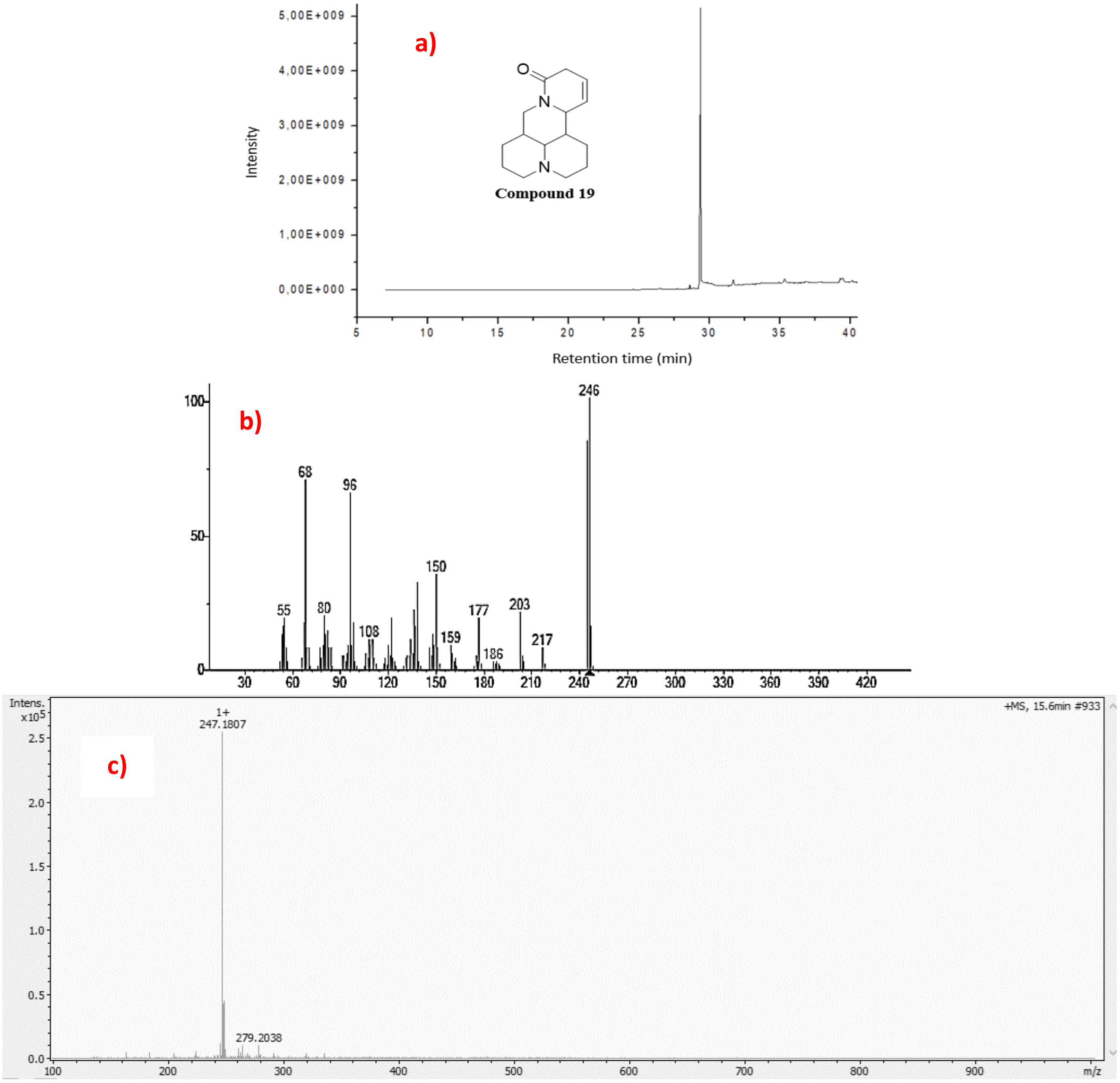


**Figure S20. a**). Chromatographic profile obtained by GC-MS of **19**; **b**). Mass spectra by electron impact at 70eV of **19**; **c**). Mass spectrum by HRESIMS of **19**.

### 6.3. Compound 20: (–)-oxymatrine

Compound **20** ($[\alpha]_D^{20}$ = –49, MeOH, *c* 0.01) was a white crystalline solid (10.7 mg, mp. 208-210°C), positive for Dragendorff's reagent, soluble in chloroform, methanol, and water. The analysis by GC-MS afforded an *m/z* = 264 that corresponds to the molecular formula $C_{15}H_{24}N_2O_2$. This information was confirmed by HRESIMS, and a $[M+H]^+$ = 265.1925 (calcd 265.1916) was obtained. The structure of compound **20** was confirmed by $^1$H and $^{13}$C NMR, whose signals were compared with the literature and agreed with the data reported for (–)-oxymatrine isolated from *Sophora flavescens* [19].

**$^1$H NMR** (500 MHz, $CDCl_3$) $\delta_H$ 5.07 (td, J = 9.9, 5.6 Hz, 1H), 4.37 (dd, J = 12.2, 5.2 Hz, 1H), 4.15 (t, J = 12.5 Hz, 1H), 3.16 – 3.11 (m, 2H), 3.09 – 3.05 (m, 1H), 3.05 – 3.03 (m, 1H), 3.02 – 2.99 (m, 1H), 2.77 – 2.67 (m, 1H), 2.66 – 2.58 (m, 1H), 2.45 – 2.38 (m, 1H), 2.25 – 2.20 (m, 1H), 2.19 – 2.13 (m, 1H), 2.02 (dd, J = 9.8, 3.3 Hz, 1H), 1.86 – 1.80 (m, 1H), 1.80 – 1.72 (m, 2H), 1.69 – 1.64 (m, 1H), 1.64 – 1.58 (m, 1H), 1.55 (dd, J = 7.3, 3.6 Hz, 1H), 1.53 – 1.51 (m, 2H), 1.49 (dd, J = 8.7, 4.3 Hz, 1H), 1.27 – 1.18 (m, 1H). **$^{13}$C NMR** (125 MHz, $CDCl_3$) $\delta_C$ 170.2, 69.8, 69.4, 67.3, 53.1, 42.9, 41.8, 34.7, 33.1, 28.7, 26.3, 24.9, 18.8, 17.3.

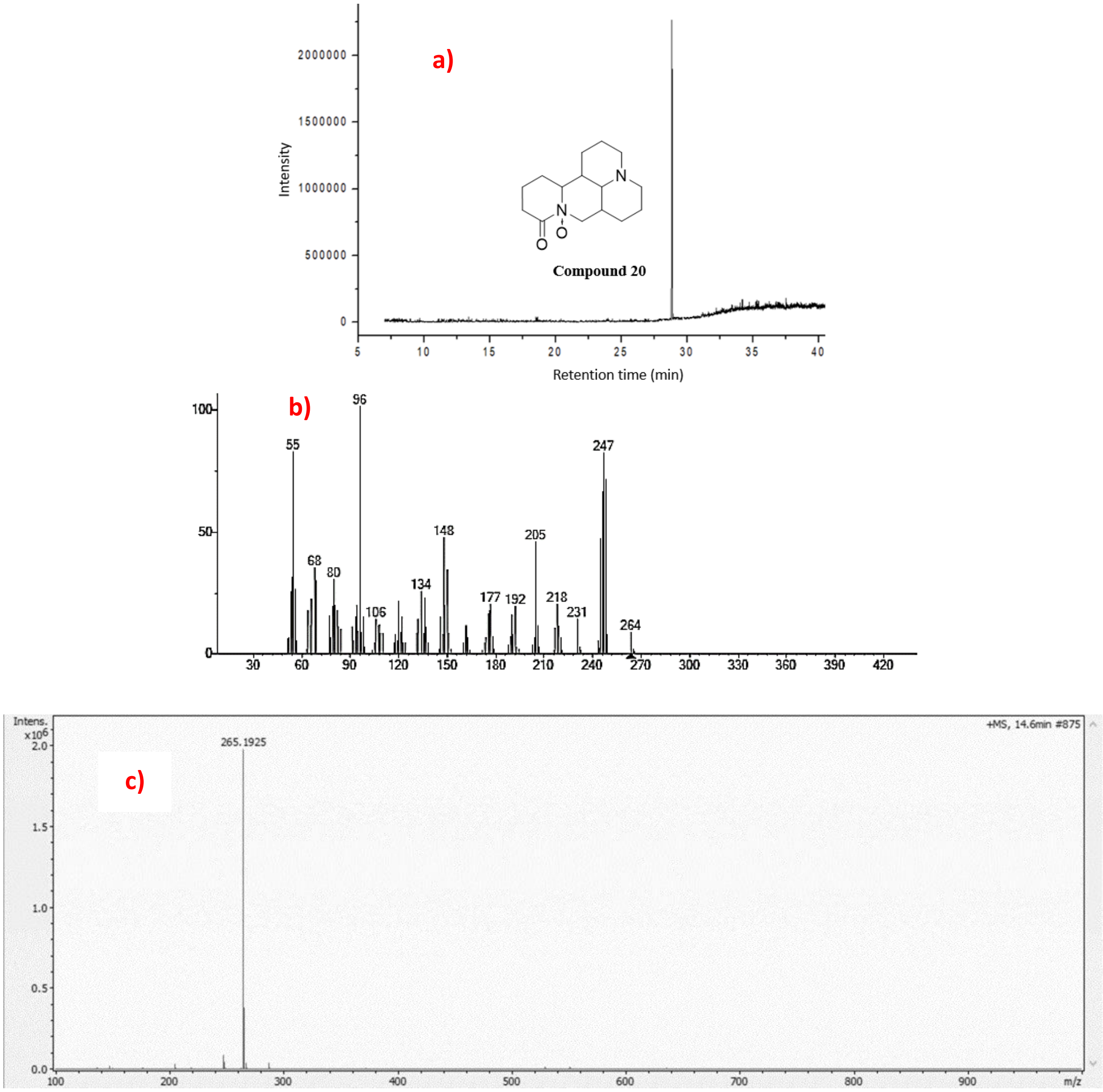


**Figure S21**. **a**). Chromatographic profile obtained by GC-MS of **20**; **b**). Mass spectra by electron impact at 70eV of **20**; **c**). Mass spectrum by HRESIMS of **20**.

# References

1. A. K. Przybył and M. Kubicki, Tetrahedron **67**, 7787 (2011).
2. Y. D. Cho and R. O. Martin, Can. J. Chem. **49**, 265 (1971).
3. M. M. Al-Azizi, M. S. Al-Said, M. M. El-Olemy, E. A. Sattar, and A. S. Khalifa, Arch. Pharm. Res. **17**, 393 (1994).
4. J. Kurek, B. Jasiewicz, and A. Katrusiak, ChemRxiv **3**, 1 (2017).
5. T. Borowiak, I. Wolska, W. Wysocka, and T. Brukwicki, J. Mol. Struct. **753**, 27 (2005).
6. W. M. Gołęlebiewski, Magn. Reson. Chem. **24**, 105 (1986).
7. W. M. Golebiewskl and I. D. Spenser, Can. J. Chem. **63**, 716 (1985).
8. R. Kolanoś, W. Wysocka, and T. Brukwicki, Tetrahedron **59**, 5531 (2003).
9. V. Galasso, F. Asaro, F. Berti, B. Kovač, I. Habuš, and A. Sacchetti, Chem. Phys. **294**, 155 (2003).
10. T. Borowiak, M. Kubicki, W. Wysocka, and A. Przybył, J. Mol. Struct. **442**, 103 (1998).
11. T. Brukwicki, W. Wysocka, and B. Nowak-Wydra, Can. J. Chem. **72**, 193 (1994).
12. D. S. Rycroft, D. J. Robins, and I. H. Sadler, Magn. Reson. Chem. **30**, S15 (1992).
13. D. Gueyrard, R. T. Tlegenov, S. Steinbruckner, B. Perly, and P. Rollin, J. Sulfur Chem. **31**, 493 (2010).
14. A.-L. Sagen, J. Gertsch, R. Becker, J. Heilmann, and O. Sticher, Phytochemistry **61**, 975 (2002).
15. K. M. Turdybekov, I. V. Kulakov, D. M. Turdybekov, and A. S. Mahmutova, Russ. J. Gen. Chem. **87**, 2493 (2017).
16. D. J. Robins and D. S. Rycroft, Magn. Reson. Chem. **30**, 1125 (1992).
17. T. Brukwicki, A. Przybyl, W. Wysocka, and J. Sośnicki, Tetrahedron **55**, 14501 (1999).
18. W. Wysocka, A. Przybył, and T. Brukwicki, Monatshefte Für Chemie **125**, 1267 (1994).
19. G. Y. Bai, D. Q. Wang, C. H. Ye, and M. L. Liu, Appl. Magn. Reson. **23**, 113 (2002).
20. Shakhnoza S. Azimova; Marat S. Yunusov., *Natural Compounds: Alkaloids* (Springer, New York, NY, 2013).
